# Cytoarchitecture of neurogenic niche and neuroblast clusters in the postnatal microminipig brain

**DOI:** 10.1016/j.stemcr.2026.102893

**Published:** 2026-04-16

**Authors:** Daijiro Kojima, Masato Sawada, Taisei Ishimaru, Nodoka Ito, Shinichiro Tateyama, Kazuhide Adachi, Hiroaki Kawaguchi, Noriaki Satake, Vicente Herranz-Pérez, José Manuel García-Verdugo, Yuichi Hirose, Nobuhiko Ohno, Naoko Kaneko, Kazunobu Sawamoto

**Affiliations:** 1 Department of Developmental and Regenerative Neurobiology, Institute of Brain Science, Nagoya City University Graduate School of Medical Sciences, Nagoya 467-8601, Japan; 2 Department of Neurosurgery, Fujita Health University, Toyoake 470-1192, Japan; 3 Division of Neural Development and Regeneration, National Institute for Physiological Sciences, Okazaki 444-8585, Japan; 4 Laboratory of Neuronal Regeneration, Graduate School of Brain Science, Doshisha University, Kyotanabe 610-0394, Japan; 5 Laboratory of Veterinary Pathology, Kitasato University School of Veterinary Medicine, Towada 034-8628, Japan; 6 Laboratory of Veterinary Histopathology, Joint Faculty of Veterinary Medicine, Kagoshima University, Kagoshima 890-0065, Japan; 7 Fuji Micra Inc., Fujinomiya 418-0005, Japan; 8 Laboratory of Comparative Neurobiology, Cavanilles Institute, University of Valencia, CIBERNED–ISCIII, 46980 Valencia, Spain; 9 Department of Cell Biology, Functional Biology and Physical Anthropology, University of Valencia, CIBERNED–ISCIII, 46100 Burjassot, Spain; 10 Department of Anatomy, Division of Histology and Cell Biology, Jichi Medical University, School of Medicine, Shimotsuke 329-0498, Japan; 11 Division of Ultrastructural Research, National Institute for Physiological Sciences, Okazaki 444-8585, Japan

**Keywords:** neural stem cells, postnatal neurogenesis, neuronal migration, microminipig, ventricular-subventricular zone

## Abstract

The ventricular-subventricular zone (V-SVZ) is the largest neurogenic niche in the postnatal mammalian brain, but its organization and migratory dynamics remain poorly understood in gyrencephalic species. Here, we provide ultrastructural and three-dimensional characterization of the V-SVZ neuroblasts in postnatal microminipigs, the smallest pig strain with unique advantages for experimental neuroscience. Transmission electron microscopy revealed developmental changes in cell composition and cytoarchitecture, with migratory neuroblasts consistently associated with glial cells and vasculatures. Notably, serial block-face scanning electron microscopy revealed that tier 3 neuroblasts, a gyrencephalic-specific population, formed elongated, chain-like clusters aligned along vessels, with conserved intracellular features such as polarized organelle distributions and growth cone extension. Radial glial fibers were prominent in neonates but diminished with age, suggesting a developmental shift to vascular scaffolds as primary migration guides. These findings establish microminipigs as a tractable gyrencephalic model for studying postnatal neurogenesis, offering new opportunities for translational research on brain repair.

## Highlights

• Microminipigs exhibit human-like postnatal neurogenesis in the V-SVZ
• The V-SVZ consists of three tiers with distinct neuroblast distributions
• Radial glia and vasculature shape the distinct migratory behaviors of neuroblasts
• Chain-forming neuroblasts in tier 3 show typical migratory characteristics

## Teaser

In this article, Sawamoto and colleagues report developmental changes in cell composition and cytoarchitecture of the ventricular-subventricular zone (V-SVZ) cells at the ultrastructural level in postnatal microminipigs, the smallest pig strain with unique advantages for experimental neuroscience. These findings provide insights into potential migration routes of neuroblasts from the V-SVZ to parenchymal regions in gyrencephalic brains during postnatal development.

## Introduction

The postnatal mammalian brain retains a remarkable capacity to generate new neurons. In rodents, radial glial cells, the embryonic neural stem cells, rapidly differentiate into ependymal cells and astrocytes after birth in most brain regions, whereas those in the ventricular-subventricular zone (V-SVZ) along the lateral walls of the lateral ventricles give rise to postnatal neural stem cells that continue to generate immature neurons (Chaker et al., 2024). These newborn neurons, or neuroblasts, form elongated chain-like aggregates and migrate toward their destinations under physiological and pathological conditions (Bressan and Saghatelyan, 2020; Nakajima et al., 2021). In humans, active neurogenesis occurs during the neonatal period but rapidly declines within the first 6 months of life (Sanai et al., 2004; 2011). Neuroblasts migrate not only to the OB but also to the prefrontal and entorhinal cortices (Kim et al., 2025; Nascimento et al., 2024; Paredes et al., 2016; Sanai et al., 2011). The neonatal human V-SVZ is divided into three tiers: tier 1, facing the ventricles and densely packed with cells; tier 2, containing dispersed neuroblasts; and tier 3, adjacent to the corpus callosum and containing clusters of neuroblasts (Paredes et al., 2016). Tiers 2 and 3, regions characteristic of gyrencephalic brains (Kim et al., 2025), are regarded as containing clusters of neuroblasts migrating toward their final destinations. However, the three-dimensional morphology of these clusters (e.g., spherical versus elongated chain-like structures) and their spatial relationships with surrounding tissues, which may underlie their directional migration, remain unclear.

Large animals such as primates and pigs have been used as preclinical models to investigate postnatal neurogenesis and neuronal migration in the human brain. Macaque monkeys, Old World monkeys with human-like gyrencephalic brain structures, have been used extensively (Gil-Perotin et al., 2009; Kornack and Rakic, 2001; Pencea et al., 2001), but their use is increasingly constrained by ethical considerations. Common marmosets, small New World monkeys, are advantageous due to their manageable size and shared cytoarchitectural features with the human V-SVZ; however, their brains are lissencephalic (Akter et al., 2020; Sawamoto et al., 2011). Pigs, by contrast, possess gyrencephalic brains, and postnatal V-SVZ neurogenesis as well as its alterations under pathological conditions have been reported (Alonso-Alconada et al., 2024; Jinnou et al., 2025; Morton et al., 2017; Porter et al., 2022; Torrijos-Saiz et al., 2025).

Nevertheless, conventional pigs are large (about 1.4 kg at birth and 100–300 kg as adults), making experimental interventions challenging. Minipigs, such as the Gottingen minipig (about 0.5 kg at birth and 35–45 kg as adults), have been developed to address this limitation (Ayuso et al., 2020). However, investigating postnatal neurogenesis requires tracking V-SVZ-derived neuroblasts across various brain regions and developmental stages, including adulthood, which would benefit from an even smaller gyrencephalic model.

Here, we report V-SVZ neurogenesis in the microminipig (*Sus scrofa domesticus*), a unique miniature pig strain derived from potbelly pigs and recognized as the smallest pig model (about 0.3 kg at birth and 25 kg as adults) (Sugiyama et al., 2011; Takasu et al., 2015). Transmission electron microscopy (TEM) revealed developmental changes in the cellular composition of the postnatal microminipig V-SVZ and structural similarities to the primate V-SVZ. Furthermore, serial block-face scanning electron microscopy (SBF-SEM) allowed us to reconstruct the 3D morphology of individual neuroblasts and their chain-like clusters in tier 3. These observations provide insights into potential migration routes of neuroblasts from the V-SVZ to parenchymal regions in gyrencephalic brains during postnatal development.

## Results

### Developmental changes in cellular morphology and distribution in the postnatal microminipig V-SVZ

**Figure 1:**
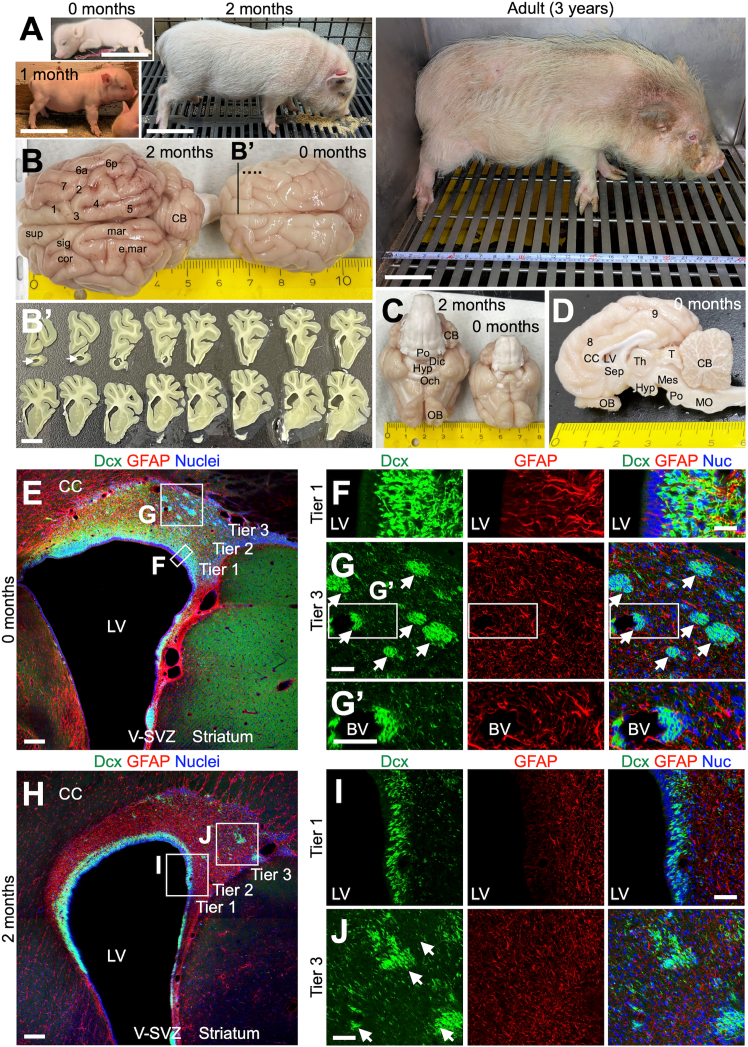
Brain structure and V-SVZ cytoarchitecture of postnatal microminipigs (A) Representative images of microminipigs at 0, 1, and 2 months and at the adult stage (3 years). (B–D) Representative images of the brain surface structure of 0-month-old and 2-month-old microminipigs. Dorsal (B), ventral (C), and sagittal (D) views of the microminipig brains are shown. Numbers in (B and D) indicate major sulci: 1, cruciate sulcus; 2, coronal sulcus; 3, ansate sulcus; 4, lateral sulcus; 5, ectolateral sulcus; 6a, anterior suprasylvii sulcus; 6p, posterior suprasylvii sulcus; 7, sulcus naris; 8, cingulate sulcus; 9, splenial sulcus. sup, superior frontal gyrus; sig, sigmoid gyrus; cor, coronal gyrus; mar, marginal gyrus; e.mar, ectomarginal gyrus. OB, olfactory bulb; Och, optic chiasm; Hyp, hypothalamus; Dic, diencephalon; CC, corpus callosum; LV, lateral ventricle; Sep, septum; Th, thalamus; T, tectum; Mes, mesencephalon; Po, pons; MO, medulla oblongata; CB, cerebellum. Representative images of coronal brain sections in 0-month-old microminipigs are shown in (B′). Arrows (B′) indicate lateral ventricles extending into the OB. (E–J) Representative images of the coronal V-SVZ sections in 0-month-old (E–G′) and 2-month-old (H–J) microminipigs stained for Dcx (green) and GFAP (red). Nuclei were stained with Hoechst 33342 (blue). Boxed areas in (E) and (H) are enlarged in (F and G) and (I and J), respectively. A hole wrapped with GFAP+ processes in (G′) is a putative blood vessel. Arrows (G and J) indicate clusters of Dcx+ neuroblasts in tier 3. LV, lateral ventricle; V-SVZ, ventricular-subventricular zone; CC, corpus callosum. Scale bars: 10 cm in (A); 1 cm in (B′); 200 μm in (E and H); 50 μm in (F, G, G′, I, and J).

**Figure 2:**
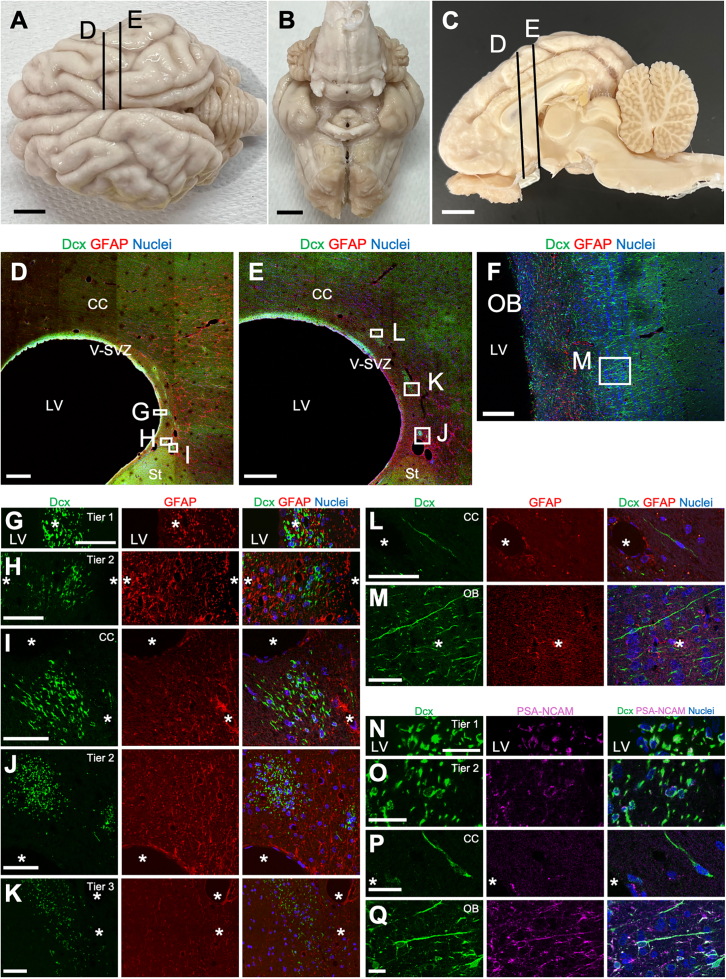
Brain structure and V-SVZ cytoarchitecture of an adult microminipig (A–C) Representative images of the brain surface structure of a 36-month-old microminipig. Dorsal (A), ventral (B), and sagittal (C) views are shown. Numbers (A and C) indicate major sulci: 1, cruciate sulcus; 2, coronal sulcus; 3, ansate sulcus; 4, lateral sulcus; 5, entolateral sulcus; 6a, anterior suprasylvii sulcus; 6p, posterior suprasylvii sulcus; 7, sulcus naris; 8, cingulate sulcus; 9, splenial sulcus. sup, superior frontal gyrus; sig, sigmoid gyrus; cor, coronal gyrus; mar, marginal gyrus; e.mar, ectomarginal gyrus. (D–M) Confocal images of the coronal brain sections at different rostrocaudal levels (indicated in A and/or C) immunostained for Dcx (green) and GFAP (red). Nuclei were stained with Hoechst 33342 (blue). Boxed areas around the LV (D and E) and the OB (F) are enlarged in (G–L) and (M), respectively. Putative blood vessels (tubular structures wrapped with GFAP+ processes in (G–M) are indicated with asterisks. (N–Q) Confocal images of the V-SVZ (N and O), CC (P), and OB (Q) immunostained for Dcx and PSA-NCAM. Nuclei were stained with Hoechst 33342. OB, olfactory bulb; Och, optic chiasm; Hyp, hypothalamus; Dic, diencephalon; CC, corpus callosum; LV, lateral ventricle; Sep, septum; Th, thalamus; T, tectum; Mes, mesencephalon; Po, pons; MO, medulla oblongata; CB, cerebellum; V-SVZ, ventricular-subventricular zone. Asterisks: blood vessels. Scale bars: 10 mm in (A–C); 500 μm in (D and E); 200 μm in (F); 50 μm in (G–M); 20 μm in (N–Q).

Microminipigs, recognized as the smallest pig model (Sugiyama et al., 2011; Takasu et al., 2015) (Figure 1A), typically reach sexual maturity between 3 and 5 months of age and have a lifespan of 12–18 years. We examined brains from neonatal (0-month-old), juvenile (2-month-old), and adult (3-year-old) microminipigs (Figures 1B–1D and 2A–2C) and identified the major gyri and sulci as previously reported in minipigs (Bjarkam et al., 2017; Yun et al., 2011) (Figures 1B–1D and 2A–2C). Well-developed OBs were visible in ventral and sagittal views (Figures 1C, 1D, 2B, and 2C), and coronal sections showed lateral ventricles extending into the OB (Figure 1B’), similar to those in minipigs (Bjarkam et al., 2017). These observations indicate that microminipig brains are smaller but retain the structural characteristics of conventional pig brains (Bjarkam et al., 2017; Yun et al., 2011).

To assess postnatal development in the V-SVZ and OB, we stained sections from neonatal, 2-month-old, and adult animals for doublecortin (Dcx, neuroblasts), polysialylated neural cell adhesion molecule (PSA-NCAM, neuroblasts), and glial fibrillary acidic protein (GFAP, astrocytes) (Figures 1E–1J, 2D–2Q, and S1A–S1H). Similar to the cytoarchitecture of neonatal pig and human brains (Kim et al., 2025; Paredes et al., 2016; Porter et al., 2022), the dorsal V-SVZ consisted of three layers: tier 1, a dense layer of Dcx+PSA-NCAM+ neuroblasts adjacent to the lateral ventricles; tier 2, containing dispersed Dcx+PSA-NCAM+ neuroblasts; and tier 3, containing clusters of Dcx+PSA-NCAM+ neuroblasts adjacent to the corpus callosum (Figure S1G). Hereafter, we mainly used an anti-Dcx antibody to visualize neuroblasts in the microminipig V-SVZ.

In tier 1, at 0 months, many Dcx+ neuroblasts were distributed up to 150 μm from the ventricular surface (Figures 1E and 1F) and were closely associated with GFAP+ radial fibers. By 2 months, tier 1 had thinned, with fewer Dcx+ neuroblasts, and radial fibers were replaced by meshwork-like GFAP+ fibers (Figures 1H and 1I). Similar age-dependent changes were also observed in the core of the OB (Figures 2F and S1). In adults, despite reduced thickness, Dcx+ cells persisted in the astrocytic fiber meshwork, particularly in the dorsomedial V-SVZ across rostrocaudal levels (Figures 2D, 2E, and 2G). To examine proliferative activity in neuroblasts in the adult V-SVZ, brain sections were immunostained for the proliferation marker Ki67 in addition to Dcx. Very few Ki67+ cells weakly expressing Dcx were detected in tier 1 (Figure S2A), and no such cells were observed in tier 2 or 3, indicating that proliferative neuroblasts are rare in the adult microminipig V-SVZ. Nevertheless, Dcx+ cells were continuously distributed throughout the microminipig V-SVZ even in adulthood, in contrast to human brains.

In tiers 2 and 3, large Dcx+ clusters were observed at birth, some associated with putative blood vessels surrounded by GFAP+ processes (Figures 1G and G'), as reported previously in neonatal pig and human brains (Paredes et al., 2016; Porter et al., 2022). By 2 months, these clusters had decreased in number, and their connections had become looser (Figure 1J), with GFAP+ processes extending between neuroblasts, suggesting that they had separated into smaller clusters.

In adults, Dcx+ cell clusters were still observed in tiers 2 and 3 and in the corpus callosum (Figures 2H–2K), in contrast to human brains (Paredes et al., 2016). GFAP+ processes were extensively interposed between neuroblasts rather than surrounding the clusters, and direct neuroblast-neuroblast contacts were occasionally observed, suggesting age-dependent changes in neuroblast and astrocyte interactions. While some Dcx+ cells co-express oligodendrocyte lineage marker Olig2 in the postnatal neocortex in common marmosets (Akter et al., 2020), Dcx+ cells in the adult microminipig brain were co-labeled with the migrating neuroblast marker PSA-NCAM (Figures 2N and 2O) but not with GFAP, Olig2, or the microglial marker Iba1 (Figures 2G–2M and S2B–S2E′), supporting the possibility that these cells were neuroblasts. PSA-NCAM immunoreactivity in neuroblasts was significantly lower in the corpus callosum than in tier 2 and the OB (Figures 2P, 2Q, and S2F), suggesting region-dependent migratory activity. Together, these findings indicate that neuroblast migration persists in the adult microminipig brain, although the mode of migration differs from that observed during postnatal development.

### Ultrastructural architecture of tiers 1 and 2 in the microminipig V-SVZ at birth

**Figure 3:**
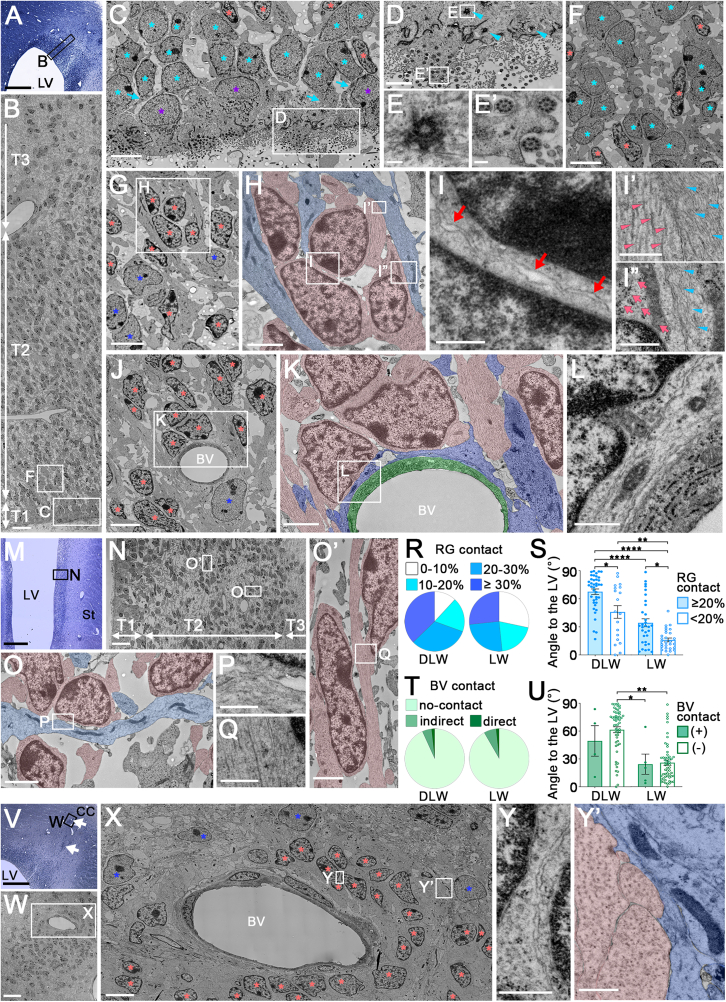
TEM of the V-SVZ in 0-month-old microminipigs (A–L) Tiers 1–2 in the dorsolateral wall (DLW). Toluidine blue-stained semi-thin section (A) and low magnification TEM (B). In tier 1 (C–E), ependymal cells with 9 + 2 cilia (E′), radial glial (RG) extending apical processes (C, cyan arrows), single neuroblasts, and 9 + 0 primary cilia (D, arrowheads; E) were observed. RGs were also present in the inner one-third of tier 2 (F). In outer tier 2 (G–L), small neuroblast clusters (H, pink) contacted RG processes (H, cyan). Neuroblasts showed narrow intercellular spaces (I, red arrows), microtubule-rich processes (I′, pink arrowheads), and scant cytoplasm with many free ribosomes (I″, pink arrows). RG processes contained intermediate filaments (I′ and I″, cyan arrowheads). A neuroblast cluster (J and K, pink) contacted a blood vessel encased by astrocytic processes (K, green: endothelium, blue: astrocytes). Higher magnification images are from boxed areas in B (C and F), C (D), D (E and E′), G (H), H (I–I″), J (K), and K (L). (M–Q) Tiers 1 and 2 in the lateral wall (LW). Semi-thin section (M) and low-magnification TEM (N) show the tiered structure. In tier 2, neuroblasts (O and O′, pink) contacted RG processes (O, cyan) or aligned along other neuroblast processes (O′, pink). Higher magnification images are from boxed areas in M (N), N (O and O′), O (P), and O′ (Q). (R) Percentage of neuroblast membrane in contact with RG (DLW: *n* = 59 cells, LW: *n* = 60 cells). (S) Nuclear axis angle relative to the ventricular surface, grouped by RG contact (≥20% vs. <20%) (DLW, ≥20%: *n* = 40 cells, <20%: *n* = 19 cells; LW, ≥20%: *n* = 32 cells, <20%: *n* = 28 cells, Steel-Dwass test). Bar graph indicates mean ± SEM. (T) Percentage of neuroblast membrane contacting blood vessels (DLW: *n* = 59 cells, LW: *n* = 60 cells). (U) Nuclear axis angle with (+) or without (−) vascular contact (DLW, +: *n* = 4 cells, −: *n* = 55 cells; LW, +: *n* = 5 cells, −: *n* = 55 cells, Steel-Dwass test). Bar graph indicates mean ± SEM. (V–Y′) Large neuroblast clusters with blood vessels in tier 3. Semi-thin section (V, arrows) and TEM (W–Y′) show densely packed neuroblasts with minimal intercellular spaces (Y), surrounded by astrocytic processes (Y′, blue). Higher magnifications from boxed areas in V (W), W (X), and X (Y and Y′). Color-coded asterisks: purple, ependymal cells; cyan, radial glia; pink, neuroblasts; blue, astrocytes. LV, lateral ventricle; St, striatum; BV, blood vessel; CC, corpus callosum; T1, tier 1; T2, tier 2; T3, tier 3. *^∗^p* < 0.05, ^∗^*^∗^p* < 0.01, *^∗∗∗^p* < 0.001, *^∗∗∗∗^p* < 0.0001. Scale bars, 500 μm in (A, M, and V); 20 μm in (B, N, and W); 5 μm in (C, F, G, J, and X); 2 μm in (D, H, K, O, and O′); 1 μm in (L and Y′); 0.5 μm in (I, I′, I″, P, Q, and Y); 0.2 μm in (E and E′).

To investigate the postnatal development of V-SVZ cytoarchitecture and neuroblast migration at the ultrastructural level, we performed TEM analysis. Cell-type classification followed established criteria (Akter et al., 2020; Doetsch et al., 1997; Torrijos-Saiz et al., 2025) (Table S1). At 0 months (Figures 3A–3Y’ and S3), a thick, high-density layer up to 300 μm from the ventricular surface (tiers 1 and 2) was observed in the dorsal wall (Figures 3A–3L). Numerous extracellular gaps were present throughout this layer (Figures 3C–3F, 3G, and 3J), unlike neighboring cortical (Figures 3V–3Y′) and striatal regions (Figures S3C–S3E′). Tier 1 (30–40 μm thick; Figure 3B, T1) consisted mainly of ependymal cells with multiple motile cilia along the ventricular surface (Figures 3C, 3D, and 3E′) and radial glial cells. Most radial glial bodies were above ependymal cells, but some contacted the ventricle (Figure 3C) and bore primary cilia (Figures 3D and 3E). Sparse neuroblasts, with small elongated nuclei, smooth contours, and numerous microtubules, similar to those in rodent and primate brains (Akter et al., 2020; Doetsch et al., 1997), were interspersed among radial glia (Figure 3C). In the inner region of tier 2 (40–100 μm from ventricle), radial glial cell bodies formed small clusters (Figures 3F and S4A), consistent with features of outer radial glia. In the outer two-thirds, dense neuroblasts and radial glial processes predominated (Figures 3B and 3G–3L). Over 60% of neuroblasts here formed small clusters of 2–6 individuals (Figures 3G and 3J) with intercellular free spaces (Figure 3I, red arrows), typical of migratory neuroblasts in the V-SVZ and RMS (Doetsch et al., 1997).

To explore potential migration routes, we analyzed neuroblast contacts with putative scaffold structures and their morphological polarity. Neuroblast clusters often contacted radial glial fibers (Figures 3H, 3I′, 3I″, and 3R). Neuroblasts with more than 20% contact showed significantly larger radial migration angles than those with less than 20% (Figure 3S), suggesting that radial glia serve as scaffolds for radial migration. Few neuroblasts were associated with blood vessels (Figures 3J and 3T), separated from the vascular basal lamina by a thin layer of astrocytic processes (Figures 3K and 3L). Our data suggest that contact with blood vessels has less influence on migration orientation during the neonatal stage compared with radial glial processes (Figure 3U). These findings suggest that in the dorsolateral wall, neuroblasts mainly migrate radially along radial glial processes toward the neocortex.

In tier 2 of the lateral wall (Figures 3M–3Q), some small neuroblast clusters remained associated with radial glial processes (Figures 3N, 3O, 3P, S4B, and S4B′), whereas many neuroblasts exhibited elongated nuclei and microtubule-rich processes oriented parallel to the ventricular surface. These cells were frequently aligned with neighboring neuroblasts, consistent with tangential migration (Figures 3O’, 3Q, and S4B–S4B″). Quantification showed that neuroblasts with close contact with radial glia had significantly larger radial orientation angles than those with less contact, even in the lateral wall, although angles were significantly smaller than those in the dorsolateral wall (Figure 3S). Vascular association was rare and did not affect orientation (Figures 3T and 3U). Taken together, in the lateral wall, neuroblasts predominantly migrate tangentially along the ventricle, with radial glial contact still modulating orientation.

### Ultrastructure of neuroblast clusters in tier 3 of the microminipig V-SVZ at birth

In contrast to tiers 1 and 2, tier 3, extending toward the white matter, was densely populated with neurons, glial cells, and their processes, although myelin sheaths were still sparse and thin at this age (Figures 3V–3Y′). Consistent with previous reports in human infants and piglets (Kim et al., 2025; Paredes et al., 2016; Porter et al., 2022; Torrijos-Saiz et al., 2025), several large clusters, occasionally containing more than 100 neuroblasts, were observed (Figures 3V and 3W). Neuroblasts within these clusters were tightly packed with minimal intercellular spaces (Figures 3X–3Y’ and S3A–S3B′). To assess the spatial extent of these clusters, we examined their morphology in serial semi-thin sections spanning >50 μm rostrocaudally. All 16 identified clusters were organized around cross-sections of small blood vessels ensheathed by astrocytic endfeet (Figures S3B and S3E′), extending across multiple sections. This organization is consistent with neuroblast migration along the rostrocaudal axis using vascular scaffolds. Similar clusters were also seen in the medial striatum adjacent to the lateral V-SVZ (Figures S3C–S3E′). Astrocytes and their extensive processes surrounded the clusters (Figures 3X and 3Y′), but only a few thin astrocytic protrusions were within clusters (Figure S3A), resembling astrocytic tunnels in the RMS (Lois et al., 1996).

Taken together, these observations indicate that neonatal neuroblasts show region-specific modes of migration. Near the ventricle, a loose tissue with many spaces, they form small clusters migrating radially along radial glial processes or tangentially along each other. In contrast, in dense parenchyma with few spaces, they aggregate into large clusters migrating along blood vessels ensheathed by astrocytic endfeet. This suggests that the microenvironment, including radial glia or vasculature, shapes distinct migratory behaviors depending on location.

### Ultrastructural architecture of the V-SVZ in 2-month-old microminipigs

**Figure 4:**
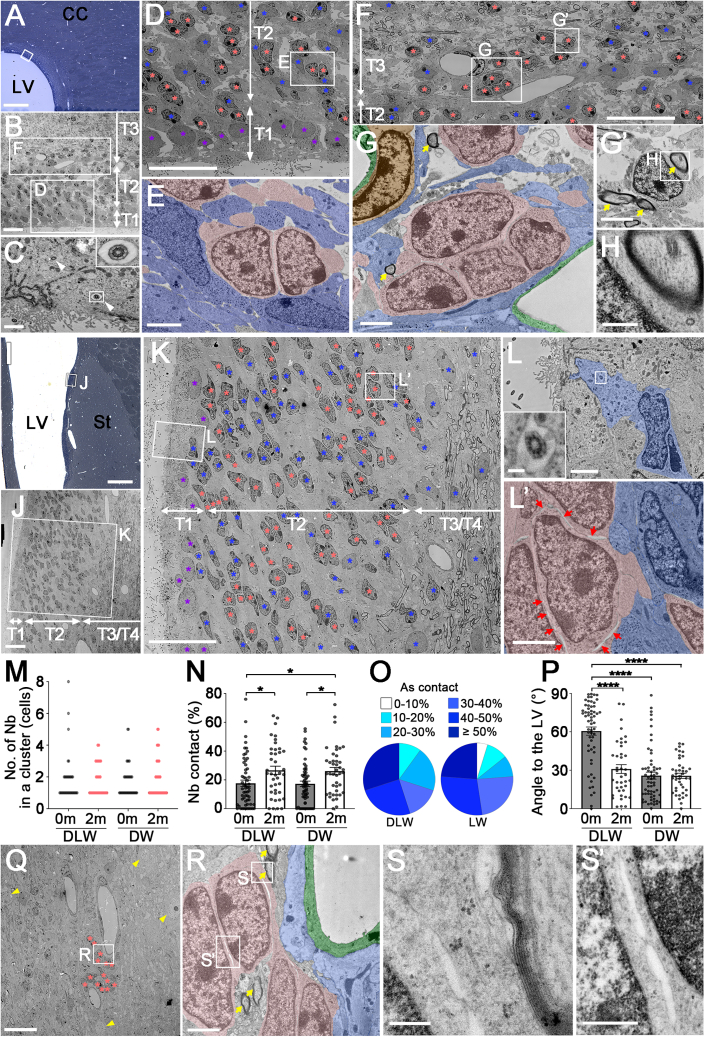
TEM of the V-SVZ in 2-month-old microminipigs (A–H) Tiers 1–3 in the dorsolateral wall (DLW). Toluidine blue-stained semi-thin section (A) and low-magnification TEM (B) show the tiered structure. In tier 1 (C and D), 9 + 0 primary cilia (C, arrowheads, inset) were rare among multiciliated ependymal cells (D). Tier 2 (D and E) contained many astrocytes and neuroblasts (single cells or small clusters) in close association (E, pink: neuroblasts, blue: astrocytes). In tier 3 (F–H), a neuroblast cluster contacted blood vessels (G, pink: neuroblasts, green: endothelium, orange: pericyte, blue: astrocytes). Neuroblasts frequently contacted myelinated axons (G–G′, yellow arrows; H). Higher magnification images are from boxed areas in B (F and D), D (E), F (G and G′), and G′ (H). (I–L′) Tiers 1–4 in the lateral wall (LW). Semi-thin section (I) and low-magnification TEM (J) show the tiered structure. In tier 1, ependymal cells lined the ventricular surface (K), and a type B1 astrocyte with an apical protrusion and 9 + 0 primary cilium was observed (L, inset). Astrocytes (single or clustered) were distributed throughout tiers (K) and closely associated with neuroblasts (L′, blue, astrocytes; pink, neuroblasts; red arrows: intercellular spaces). Higher magnification images are from boxed areas in I (J), J (K), and K (L and L′). (M) Dot plots showing the number of neuroblasts per cluster at 0 months (0 m) and 2 months (2 m) (DLW, 0 m: *n* = 68 cells, 2 m: *n* = 72 cells; LW, 0 m: *n* = 63 cells, 2 m: *n* = 72 cells). (N) Percentage of neuroblast membrane in contact with other neuroblasts at 0 m and 2 m (DLW, 0 m: *n* = 59 cells, 2 m: *n* = 40 cells; LW, 0 m: *n* = 60 cells, 2 m: *n* = 42 cells, Steel-Dwass test). Bar graph indicates mean ± SEM. (O) Percentage of neuroblast membrane in contact with astrocytes at 2 m (DLW: *n* = 40 cells; LW: *n* = 42 cells). (P) Nuclear axis angle relative to the ventricular surface at 0 m and 2 m (DLW, 0 m: *n* = 59 cells, 2 m: *n* = 40 cells; LW, 0 m: *n* = 60 cells, 2 m: *n* = 41 cells, Steel-Dwass test). Bar graph indicates mean ± SEM. (Q–S′) Neuroblast clusters in the striatum. Low magnification image (Q) shows a cluster surrounded by myelinated axons and striatal neurons (yellow arrowheads). Neuroblasts contacted myelinated axons and astrocytic endfeet on blood vessels (R, pink: neuroblasts, green: endothelium, blue: astrocytes, yellow arrows: myelinated axons). Higher magnification images are from boxed areas in Q (R) and R (S and S′). Color-coded asterisks: purple, ependymal cells; pink, neuroblasts; blue, astrocytes. LV, lateral ventricle; St, striatum; CC, corpus callosum; T1, tier1; T2, tier2; T3, tier3. *^∗^p* < 0.05, *^∗∗∗∗^p* < 0.0001. Scale bars, 500 μm in (A and I); 20 μm in (B, D, F, J, K, and Q); 2 μm in (E, G, G′, L, L′, and R); 1 μm in (C); 0.5 μm in (H and S′); 0.2 μm in (L) inset, (S).

By 2 months, tiers 1 and 2 had shrunk to less than 80 μm in thickness in both the dorsolateral wall (Figures 4A–4H) and the lateral walls (Figures 4I–4L’ and S4C). Among ependymal cells lining the ventricle, type B1 astrocytes with an apical protrusion bearing a primary cilium (Figures 4L and 4L′) were rarely observed. Radially oriented glial nuclei and processes were absent; instead, astrocytes with irregular contours and light cytoplasm containing intermediate filaments were distributed (Figure 4D). Neuroblasts typically formed small clusters of 2–5 cells (Figures 4D–4G). While average cluster size was similar between the 0- and 2-month groups (Figure 4M), the percentage of plasma membranes contacting other neuroblasts was increased significantly in the latter group (Figure 4N). Despite these contacts, intercellular free spaces indicative of active migration remained visible between membranes (Figure 4L’). Individual and clustered neuroblasts were extensively associated with astrocytic cell bodies/processes (Figures 4E, 4L′, 4O, and S4C).

To examine the effect of the age-dependent changes in V-SVZ composition on neuroblast migration direction, we compared their orientation relative to the ventricular wall (Figure 4P). In the 2-month dorsolateral wall, orientation was diverse, with only 10% of nuclei oriented radially (>60°) toward the corpus callosum. The mean angle was significantly smaller than at birth, when about 64% were oriented radially. In the lateral wall, the largely parallel orientation was similar between ages; however, radially oriented neuroblasts observed at birth were absent at 2 months. Thus, within 2 months, radial migration toward adjacent parenchyma markedly decreased, coinciding with the loss of radial glia.

In tier 3 regions, many axons with thick myelin sheaths were present, and the boundary with the corpus callosum was indistinct in the lateral wall (Figure 4Q). Large tightly packed clusters seen in neonates were absent; neuroblasts appeared in small clusters or individually (Figures 4F–4H and 4Q–4S′). These cells were surrounded by astrocytic processes, and occasionally contacted blood vessels via astrocytic endfeet (Figures 4G and 4R) or directly with myelinated axons (Figure 4G, 4H, 4R, and 4S). Even in dense parenchyma, intercellular free spaces between neuroblasts were still evident (Figure 4S’), suggesting that migration in parenchymal regions (corpus callosum and striatum) was reduced but still observed along blood vessels and axons.

### Three-dimensional morphology of individual neuroblasts in tier 3

**Figure 5:**
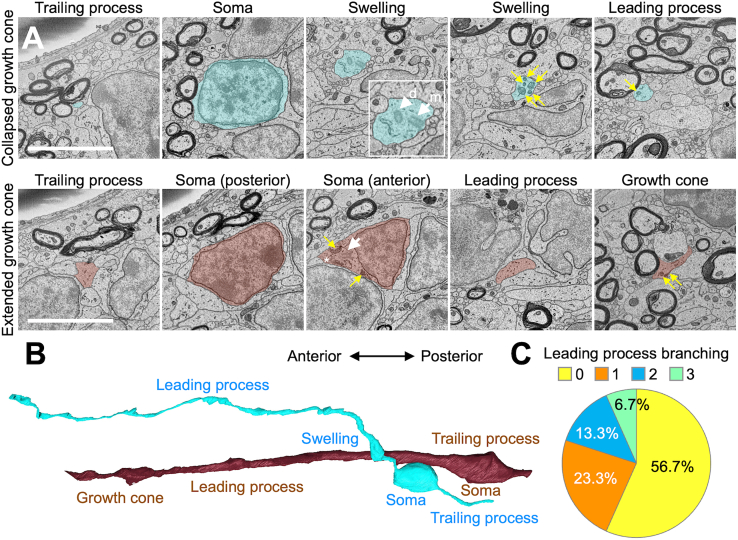
Three-dimensional morphology of individual neuroblasts in blood vessel-associated clusters in tier 3 (A) Representative SBF-SEM images of migratory neuroblasts with (bottom) or without (top) the growth cone in tier 3 of the V-SVZ in 2-month-old microminipigs. The migratory neuroblasts consist of the trailing process, soma, swelling (occasional), leading process, and the growth cone. White and yellow arrows indicate centrosomes and mitochondria, respectively. Asterisk [bottom, soma (anterior)] indicates the Golgi apparatus. Swelling in the neuroblast with a collapsed growth cone (top, swelling) is magnified in the right bottom box (m, mother centriole; d, daughter centriole). (B) Representative 3D reconstructions of migratory neuroblasts shown in (A). (C) Proportion of neuroblasts with unbranched and branched leading processes. Scale bars, 10 μm.

Clusters of Dcx+ neuroblasts in tier 3 are a unique feature of gyrencephalic animals, including pigs and humans (Kim et al., 2025; Paredes et al., 2016; Porter et al., 2022; Torrijos-Saiz et al., 2025), but whether these cells show typical migratory morphology remains unclear. To address this issue, we imaged more than 2,000 consecutive 80-nm-thick sections from 2-month-old microminipigs using SBF-SEM. In blood vessel-associated neuroblast clusters, neuroblasts showed unipolar or bipolar smooth cell contours, dark cytoplasm, and a small Golgi apparatus (Figures 5A and 5B), consistent with features reported in mice (Matsumoto et al., 2019).

Among clustered neuroblasts, 56.7% extended an unbranched leading process resembling that in mice; the rest of the cells had one (23.3%), two (13.3%), or three (6.7%) branches in their leading process (Figures 5B and 5C). Growth cones, either extended or collapsed, were seen at the tip (Nakajima et al., 2024) (Figure 5B, brown). Mitochondria, Golgi apparatus, and paired centrioles accumulated in the proximal leading process or in a swelling, as in mice (Matsumoto et al., 2019) (Figure 5A; yellow arrows, asterisks, and white arrows, respectively). These results indicate that tier 3 neuroblasts in postnatal microminipigs retain typical migratory morphology.

### Three-dimensional morphology of neuroblast clusters in tier 3

**Figure 6:**
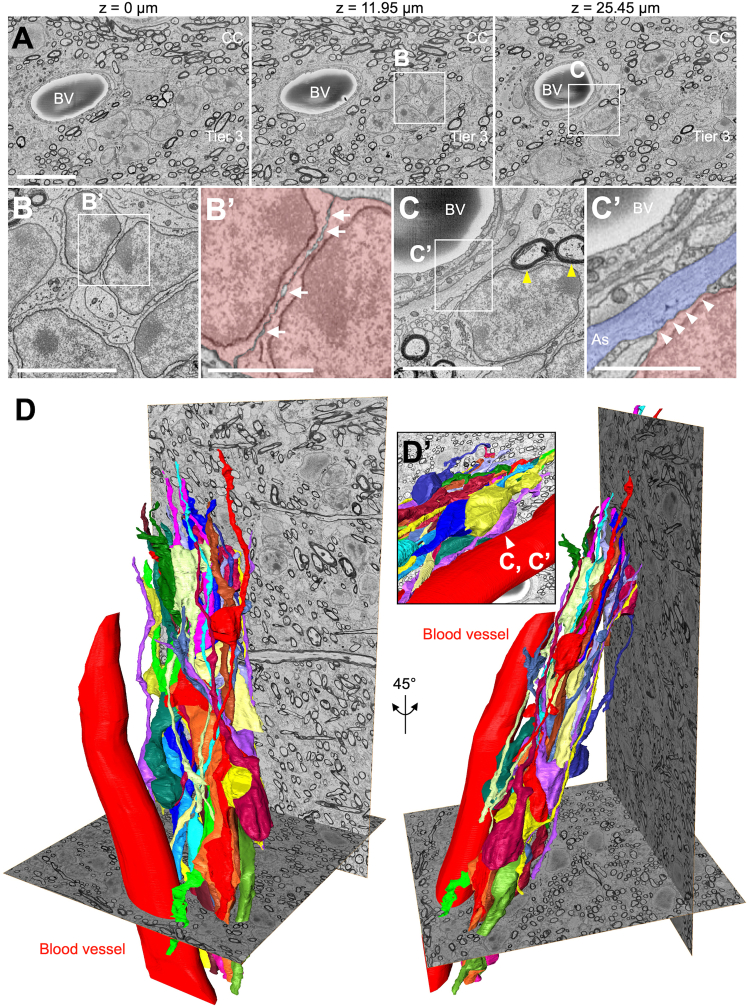
Three-dimensional morphology of blood vessel-associated neuroblast clusters in tier 3 (A–C′) Representative SBF-SEM images of blood vessel-associated clusters of migratory neuroblasts in tier 3 of the V-SVZ in 2-month-old microminipigs. Boxed areas in (A) are magnified in (B, C). Boxed areas in (B) and (C) are further magnified in (B′) and (C′), respectively. Arrows (B′) indicate intercellular free spaces at the contact site of two neuroblasts. Yellow and white arrowheads (C and C′) indicate neuroblast contact to myelinated axons and an astrocytic process (As), respectively. BV, blood vessel. (D) Three-dimensional reconstructions of clusters of migratory neuroblasts and an associated blood vessel shown in (A–C′). Contact site between neuroblast and blood vessel (C and C′) is shown in (D′). Scale bars: 10 μm in (A); 5 μm in (B and C); 2 μm in (B′ and C′).

The ultrastructural morphology of cluster-forming neuroblasts in tier 3 was analyzed using serial SBF-SEM images. As seen in immunostaining and TEM, some neuroblasts within clusters contacted blood vessels via thin astrocytic processes containing intermediate filaments (Figures 6A, 6C, and 6C′). These clusters also interacted with both myelinated and non-myelinated axons (Figures 6A and 6C). Intercellular free spaces between neuroblasts (Figures 4R and 4S′) were consistently observed (Figures 6A and 6B′), supporting the idea that these cells migrate actively within the cluster.

To determine overall cluster shape, we reconstructed clusters’ 3D morphology (Figure 6D). Clusters were not spherical aggregates but elongated chain-like structures composed of neuroblasts with migratory morphology (Figure 6D). Because such clusters were frequently associated with blood vessels (Figures 1 and 4), we next examined the spatial relationship between them. Neuroblasts were partially in contact with vessel walls (Figures 6C, 6C’, and 6D’) and aligned parallel to them (Figure 6D), suggesting that blood vessels may serve as migratory scaffolds, similar to blood vessel-associated clusters observed in the same region at birth (Figures 3V–3X).

**Figure 7:**
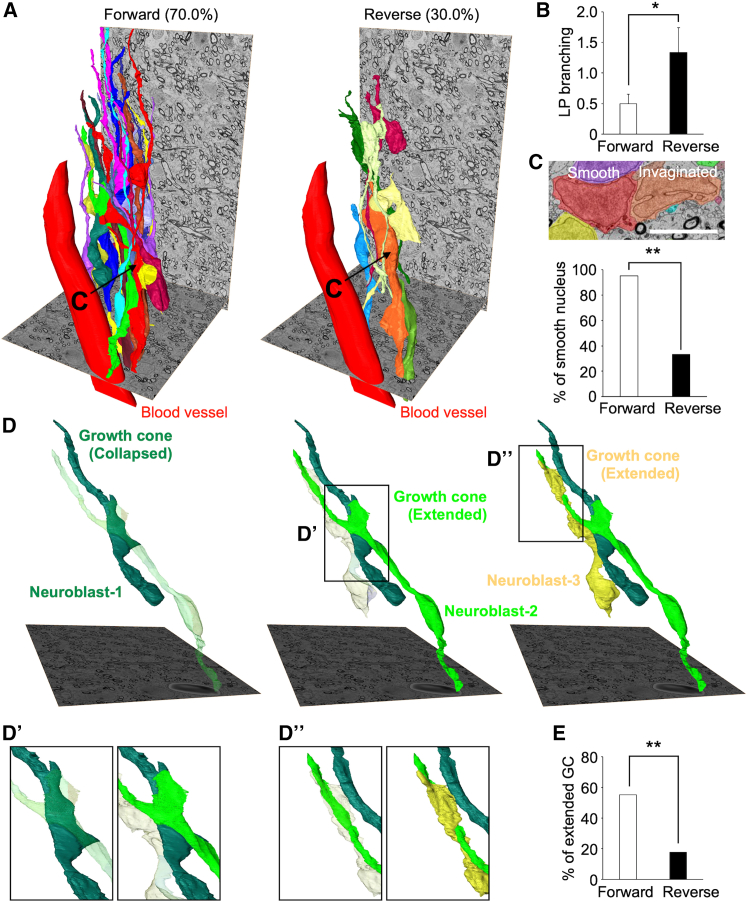
Morphology of growth cone, leading process, and nucleus in cluster-forming neuroblasts in tier 3 (A) Three-dimensional reconstructions of forward- and reverse-migrating neuroblasts. Nuclear morphology of the neuroblasts indicated by arrows is shown in (C). (B) Number of leading process branching in forward- and reverse-migrating neuroblasts (unpaired *t* test). Bar graph indicates mean ± SEM. (C) Proportion of cells with smooth nucleus in forward- and reverse-migrating neuroblasts (Fisher’s exact test). Representative SBF-SEM image of cells with smooth (red) and invaginated (orange) nucleus, whose 3D morphology is shown in (A), is also shown. (D) Representative 3D reconstruction images of cluster-forming neuroblasts. Growth cones of neuroblast-2 and -3 are enlarged in (D′) and (D″), respectively. (E) Proportion of cells with extended growth cone in forward- and reverse-migrating neuroblasts (Fisher’s exact test). Scale bars, 5 μm. ^∗^*p* < 0.05, ^∗∗^*p* < 0.01.

To assess the orientation of neuroblasts within clusters, we determined the direction of the leading process in neuroblasts based on the centriole position. Quantitative analysis showed that 70.0% (21/30) of neuroblasts extended their leading process forward and 30.0% (9/30) in the reverse direction (Figure 7A), indicating directional migration. We then examined how migration direction related to the leading process and nuclear morphology. Forward-migrating neuroblasts had fewer branches in the leading process and a higher proportion of smooth nuclei than reverse-migrating cells (Figures 7B and 7C), suggesting more efficient leading process extension and subsequent somal translocation.

We further analyzed growth cone morphology in relation to migration direction. Similar to chain-forming neuroblasts in mice, microminipig neuroblasts extended their growth cone along neighboring cells in the cluster (Figures 7D–7D″). Extended growth cones were significantly more frequent in forward-migrating neuroblasts than in reverse-migrating ones (Figure 7E), suggesting active extension of the leading process during migration.

Finally, to investigate the relationship between blood vessel association and neuroblast cluster morphology, we analyzed a cluster located farther from blood vessels in tier 3 (Figure S5). In contrast to blood vessel-associated clusters, this non-associated cluster consisted of neuroblasts with irregular morphology and reduced organization (Figure S5A). At the single-cell level, neuroblasts in this cluster did not exhibit a clear relationship between migratory directionality and nuclear morphology (Figure S5C) and showed increased branching of leading processes together with reduced growth cone extension (Figures S5B and S5D). Together, these results suggest that blood vessels contribute to the directional migration of neuroblasts in chain-like clusters in tier 3.

Taken together, these results suggest that tier 3 neuroblasts form chain-like elongated clusters and migrate actively along blood vessels in postnatal microminipigs.

## Discussion

In this study, we revealed developmental changes in cell composition and cytoarchitecture of the V-SVZ cells at the ultrastructural level in postnatal microminipigs. Migratory neuroblasts were consistently associated with surrounding glial cells and blood vessels. Three-dimensional reconstruction showed that cluster-forming neuroblasts in tier 3, a population characteristic of gyrencephalic brains (Kim et al., 2025; Paredes et al., 2016), retained typical migratory morphology and assembled into elongated, chain-like aggregates aligned along blood vessels. These findings indicate that the microenvironment provides region- and age-specific scaffolds that shape neuroblast migration patterns.

Our SBF-SEM analysis further demonstrated that microminipig neuroblasts displayed conserved intracellular features associated with migration. In mice, the Golgi apparatus, centrosomes, and mitochondria accumulate in the proximal domain of the leading process or its swelling, where they regulate cytoskeletal remodeling and energy metabolism (Bressan et al., 2020; Matsumoto et al., 2019). Neuroblasts also extend a growth cone at the tip of their leading process, driving efficient movement (Nakajima et al., 2024). The observation of similar organelle distributions and growth cone morphology in microminipigs (Figure 5) suggests that common cellular machinery underlies migration across species.

Blood vessels are well established as migratory scaffolds for neuroblasts in the postnatal brain. In rodents, they guide neuroblasts along the V-SVZ-RMS-OB pathway, the corpus callosum, and toward lesion sites after stroke (Nakajima et al., 2021). In common marmosets, blood vessels also support neuroblast migration toward the cerebral cortex during postnatal development (Akter et al., 2020). In microminipigs, we observed that neuroblasts were attached to vessel walls and oriented parallel to them (Figure 6), supporting the idea that vascular scaffolds and their molecular milieu create a favorable environment for directed migration in the postnatal gyrencephalic brain.

While vascular scaffolds are used throughout life, radial glia serve as migratory guides primarily during embryonic and early postnatal periods. In mice, radial glia disappear soon after birth, but can persist or reappear following brain injury to direct neuroblasts toward the injured neocortex (Jinnou et al., 2018). In neonatal microminipigs, radially oriented neuroblasts were frequently observed in close association with radial glial fibers, suggesting that radial glia guide neuroblasts radially toward adjacent parenchymal regions, including the corpus callosum, neocortex, and striatum, during early postnatal development (Figures 1 and 3). In contrast, blood vessels appeared to preferentially guide neuroblasts along the rostrocaudal axis (Figures 3 and 6), a route that may direct cells toward the OB, which is more prominently developed in pigs than in primates. Taken together, these observations suggest that, in the postnatal brain, neuroblasts utilize either radial glial fibers or blood vessels depending on their migration direction. As radial glial fibers diminish during development, vascular scaffolds may become the predominant long-term guidance structure for neuroblast migration.

In higher mammals, including humans, outer radial glia are abundant during embryonic development and contribute to the expansion of the gyrencephalic cerebral cortex. In conventional pigs, neonatal outer radial glia become reactivated after injury, producing oligodendrocyte precursor cells that aid in functional recovery (Jinnou et al., 2025). Although further studies are required to clarify their role in microminipigs, we identified a similar population in inner-tier 2 of the neonatal V-SVZ (Figure 3), implying that radial glia in gyrencephalic species may have dual roles, as migratory scaffolds and as progenitors, supporting both brain development and repair.

Gyrencephalic species exhibit an expanded V-SVZ, in which the number of neuroblasts decreases as brain size increases during postnatal development (Kim et al., 2025; Paredes et al., 2016; Porter et al., 2022). While microminipigs share these general features, they uniquely maintain neuroblast clusters in adulthood, a property more reminiscent of lissencephalic species. Thus, microminipigs may represent an intermediate evolutionary state in which gyrencephalic brain organization coexists with partial preservation of lissencephalic-like maintenance of neuroblasts in adulthood. Dcx+ neuroblast clusters persist into adulthood, suggesting that neuroblast migration may remain active in the adult microminipig brain; however, their destinations remain unknown. Because Dcx+ neuroblasts are thought not to be newly generated in other gyrencephalic species (Bonfanti et al., 2024; La Rosa et al., 2018; Piumatti et al., 2018), it is possible that a subset of Dcx+ clusters observed in adult microminipigs (Figure 2) similarly represents immature, but not newly generated, neuroblasts.

Non-human primates, such as common marmosets and macaques, have been invaluable for elucidating postnatal human neurogenesis (Akter et al., 2020; Gil-Perotin et al., 2009; Kornack and Rakic, 2001; Pencea et al., 2001; Sawamoto et al., 2011). Ferrets have also served as a useful model for studying cortical folding, owing to their suitability for genetic manipulation (Shinmyo et al., 2022; Wang et al., 2024). However, there is a growing need for additional gyrencephalic animal models suitable for experimental manipulation. Microminipigs (Abe et al., 2018; Sugiyama et al., 2011; Takasu et al., 2015; Takeishi et al., 2018), which are smaller than previously reported miniature pigs (Ayuso et al., 2020), offer advantages for husbandry and enable surgical and pharmacological studies with greater feasibility. Establishing models of brain injury such as ischemic stroke or traumatic brain injury in microminipigs will facilitate the evaluation of regenerative strategies with direct translational potential.

In summary, our ultrastructural and 3D analyses reveal that microminipig neuroblasts retain conserved migratory machinery and undergo a developmental shift from radial glial to vascular scaffolds. These features, together with their tractable size and gyrencephalic brain, position microminipigs as a powerful preclinical model for testing strategies that enhance endogenous neurogenesis and migration after brain injury.

## Methods

### Animals

Neonatal microminipigs (*Sus scrofa domesticus*) (0–3 days old, referred to as 0 month old; 0.3–0.5 kg; *n* = 5) and juvenile microminipigs (2 months old; 5–6 kg; *n* = 5) were obtained from Fuji Micra Inc. (Shizuoka, Japan). An adult female microminipig (36 months old; 28 kg; *n* = 1) was maintained at Kagoshima University. The room was maintained in a laminar flow of filtered air at a temperature of 24 ± 3°C, a relative humidity of 50 ± 20%, and a 12-h light/dark cycle. The animal had free access to tap water and was provided a normal chow diet (NcD; Kodakara 73; Marubeni Nisshin Feed Inc., Tokyo, Japan) on a daily basis. Research was performed according to the Institutional Guidelines for Animal Experiments and in compliance with the Japanese Act on Welfare and Management of Animals (Act No. 105 and Notification No. 6).

### TEM

Brains of neonatal and 2-month-old microminipigs were fixed by transcardiac perfusion with 2.5% glutaraldehyde (GA) and 2% PFA in 0.1 M PB (pH 7.4) and postfixed in the same fixative at 4°C. 300-μm-thick coronal sections from the OB to anterior V-SVZ regions were prepared for semi-thin (2 μm thick) and ultra-thin (60–70 nm thick) sectioning. Cell types were identified as previously described (Akter et al., 2020; Doetsch et al., 1997; Torrijos-Saiz et al., 2025). Further details are provided in the supplemental information.

### SBF-SEM

SBF-SEM observation of tier 3 of the V-SVZ was performed using a Merlin scanning electron microscope (Carl Zeiss) equipped with a 3View in-chamber ultramicrotome system (Gatan). Segmentation of the cell membrane was performed using Microscopy Image Browser (Belevich et al., 2016). Three-dimensional reconstruction was performed using Amira software (Maxnet Co., Ltd, Tokyo, Japan). Further details are provided in the supplemental information.

### Statistics

All statistical analyses were two-tailed and conducted using EZR (Kanda, 2013) or GraphPad Prism version 10.5.0 (GraphPad Software, La Jolla, CA, USA). A *p* value < 0.05 was considered statistically significant. Bar graphs indicate mean ± SEM. Further details are provided in the supplemental information.

## Resource availability

### Lead contact

Requests for further information and resources should be directed to and will be fulfilled by the lead contact, Kazunobu Sawamoto (sawamoto@med.nagoya-cu.ac.jp).

### Materials availability

This study did not generate new unique reagents.

### Data and code availability

Any information required to reanalyze the data reported in this paper is available from the lead contact upon request.

## Acknowledgments

This work is dedicated to the memory of Prof. José Manuel García-Verdugo, who passed away before the submission of this paper. We thank N. Hattori, A. Imai (National Institute of Physiological Sciences), M. Tanaka, S. Nakamura (Nagoya City University), and the Research Equipment Sharing Center at 10.13039/501100008883Nagoya City University for providing technical support (JPMXS0441500024) and Sawamoto laboratory members for discussions. This work was supported by research grants from Japan Agency for Medical Research and Development (10.13039/100009619AMED) (25ym0126807, 24gm1210007, and 21bm0704033h0003 [to K.S.]); 10.13039/501100001691Japan Society for the Promotion of Science (JSPS) 10.13039/501100001691KAKENHI (19H04757, 19H04785, 18KK0213, 20H05700, and JP22H04926 [to K.S.], 20H03565, 21H05106, 23H02579, and 23K27270, [to N.K.], 18K14823, 21K06395, and 24K09660 [to M.S.], and 20K09377 [to K.A.]); Bilateral Open Partnership Joint Research Projects (14544620 [to K.S.] and JPJSBP120229939 [to N.K.]); Core-to-Core program “Neurogenesis Research & Innovation Center (NeuRIC)” (JPJSCCA20230007 to K.S.); 10.13039/501100001695JST FOREST Program (JPMJFR2146 to N.K.); Grant-in-Aid for Research at Nagoya City University (to M.S. and K.S.); Grant-in-Aid for Promotion on Co-Creative Urban Development in 10.13039/501100008883Nagoya City University (2412145 to K.S.); Grant-in-Aid for Outstanding Research Group Support Program in 10.13039/501100008883Nagoya City University grant number 2401101 (to K.S.); Cooperative Study Programs of 10.13039/501100006323National Institute for Physiological Sciences (to K.S.); the 10.13039/100009578Mizutani Foundation for Glycoscience (to K.S.); Valencian Council for Education, Culture, University and Employment (CIPROM/2023/053) (to J.M.G.-V. and V.H.-P.); and the 10.13039/100007449Takeda Science Foundation (to M.S. and K.S.).

## Author contributions

D.K., M.S., T.I., N.I., S.T., K.A., H.K., N.S., N.O., and N.K. conducted the experiments; D.K., M.S., V.H.-P., J.M.G.-V., Y.H., N.O., N.K., and K.S. analyzed the results; D.K., M.S., N.K., and K.S. wrote the manuscript.

## Declaration of interests

N.S. is the president of Fuji Micra Inc.

## Supplemental information

Document S1. Figures S1–S5, Table S1, and Method S1

Document S2. Article plus supplemental information
