# Supplementary material for: Cytoarchitecture of neurogenic niche and neuroblast clusters in the postnatal microminipig brain
Source: Stem Cell Reports. 2026 Apr 16;21(5):102893. doi: 10.1016/j.stemcr.2026.102893 (PMC13163217; doi:10.1016/j.stemcr.2026.102893)
Supplement: Document S1. Figures S1–S5, Table S1, and Method S1 [file mmc1.pdf]

Stem Cell Reports, Volume *21*

Supplemental Information

Cytoarchitecture of neurogenic niche and neuroblast clusters in the postnatal microminipig brain

Daijiro Kojima, Masato Sawada, Taisei Ishimaru, Nodoka Ito, Shinichiro Tateyama, Kazuhide Adachi, Hiroaki Kawaguchi, Noriaki Satake, Vicente Herranz-Pérez, José Manuel García-Verdugo, Yuichi Hirose, Nobuhiko Ohno, Naoko Kaneko, and Kazunobu Sawamoto

Supplementary Information

Cytoarchitecture of neurogenic niche and neuroblast clusters in the postnatal microminipig brain

Daijiro Kojima, Masato Sawada, Taisei Ishimaru, Nodoka Ito, Shinichiro Tateyama, Kazuhide Adachi, Hiroaki Kawaguchi, Noriaki Satake, Vicente Herranz-Pérez, José Manuel García-Verdugo, Yuichi Hirose, Nobuhiko Ohno, Naoko Kaneko, Kazunobu Sawamoto

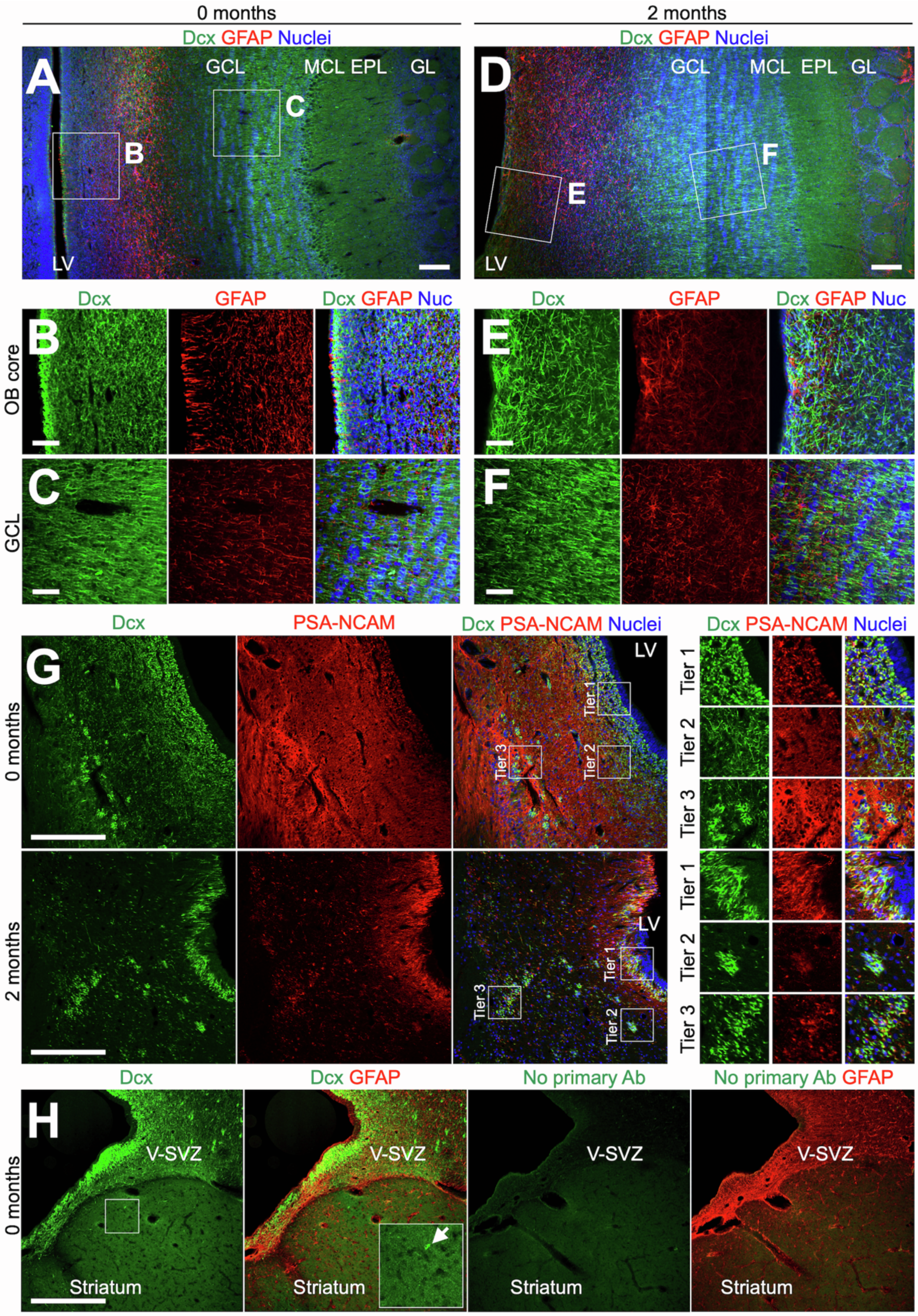

Figure S1. Cytoarchitecture of the OB in postnatal microminipigs, related to Figure 1.

A-F: Representative images of the coronal OB sections in 0-month-old (A-C) and 2-month-old (D-F) microminipigs stained for Dcx (green) and GFAP (red). Nuclei were stained with Hoechst 33342 (blue).

G: Representative images of the coronal V-SVZ sections from 0-month-old (upper panels)

and 2-month-old (lower panels) microminipigs stained for Dcx (green) and PSA-NCAM (red). Nuclei were stained with Hoechst 33342 (blue). Boxed areas are shown at higher magnification in the right panels.

H: Representative coronal sections of the V-SVZ from 0-month-old microminipigs stained for Dcx (green) and GFAP (red) (left two panels), or processed without primary antibodies (green) (right two panels). The boxed area is magnified in the second panel. The arrow indicates a Dcx+ cell in the striatum.

LV, lateral ventricle; GCL, granule cell layer; MCL, mitral cell layer; EPL, external plexiform layer; GL, glomerular layer; V-SVZ, ventricular-subventricular zone.

Scale bars: A, D, 1 cm; B, C, E, F, G, H, 200 μm.

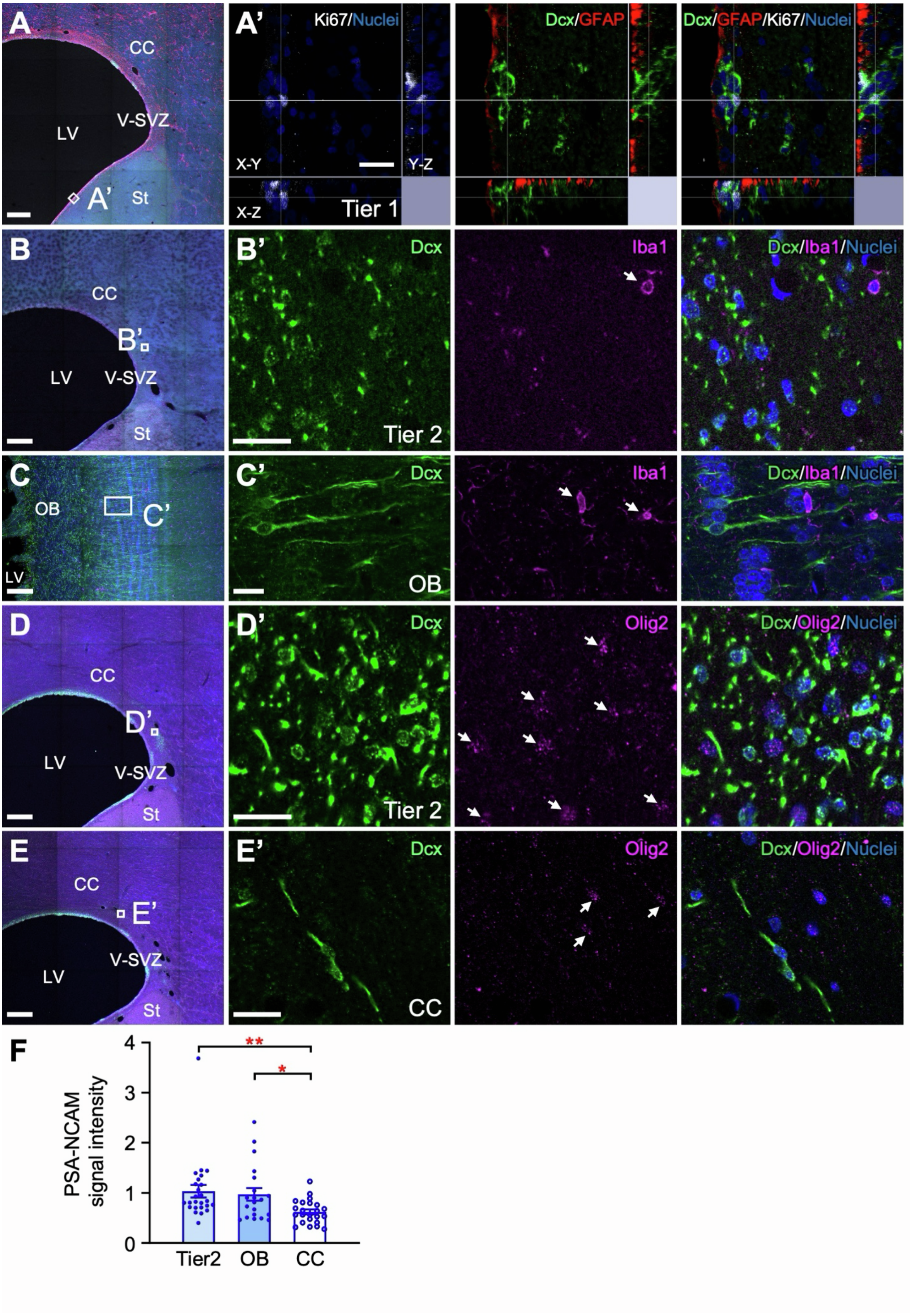

A
CC
V-SVZ
LV
A'
St
A'
Ki67/Nuclei
X-Y
Y-Z
X-Z
Tier 1
Dcx/GFAP
Dcx/GFAP/Ki67/Nuclei
B
CC
B'
LV
V-SVZ
St
B'
Dcx
Tier 2
Iba1
Dcx/Iba1/Nuclei
C
OB
C'
LV
C'
Dcx
OB
Iba1
Dcx/Iba1/Nuclei
D
CC
D'
LV
V-SVZ
St
D'
Dcx
Tier 2
Olig2
Dcx/Olig2/Nuclei
E
CC
E'
V-SVZ
LV
St
E'
Dcx
CC
Olig2
Dcx/Olig2/Nuclei
F
PSA-NCAM signal intensity
4
3
2
1
0
**
*
Tier2
OB
CC

Figure S2. Characterization of Dcx+ cells, related to Figure 2.

A: Confocal images of coronal brain sections from an adult microminipig immunostained for Dcx (green), GFAP (red), and the proliferation marker Ki67 (white). Nuclei were stained with Hoechst 33342 (blue). The boxed area is shown at higher magnification in A', with orthogonal views displayed as x-y (bottom) and x-z (right) panels.

B-C: Confocal images of the adult microminipig coronal brain sections immunostained for neuroblast marker Dcx (green) and microglial marker Iba1 (magenta). Nuclei were stained with Hoechst 33342 (blue). B shows a higher magnification image of the boxed area in A. Iba1+ cells (arrows) did not co-express Dcx in either tier 2 (A-B) or olfactory bulb (C).

D-E: Confocal images of the adult microminipig coronal brain sections immunostained for Dcx (green) and oligodendrocyte lineage marker Olig2 (magenta). Nuclei were stained with Hoechst 33342 (blue). E shows a higher magnification image of the boxed area in D. Olig2+ cells (arrows) did not co-express Dcx in either tier 2 (D-E) or corpus callosum (F). OB, olfactory bulb; CC, corpus callosum.

F: Quantification of PSA-NCAM signal intensity in Dcx+ cells located in tier 2, the corpus callosum (CC), and the olfactory bulb (OB). To normalize for background staining, the mean cytoplasmic PSA-NCAM (magenta) signal intensity of each cell, measured using Fiji, was divided by the corresponding nuclear signal intensity. PSA-NCAM signal intensity was significantly lower in Dcx+ cells in the CC compared with those in tier 2 and the OB (Kruskal-Wallis test). *$P<0.05$, **$P<0.01$.

Scale bars, 500 µm: A, B, D, E; 200 µm: C; 20 µm: A', B', C', D', E'.

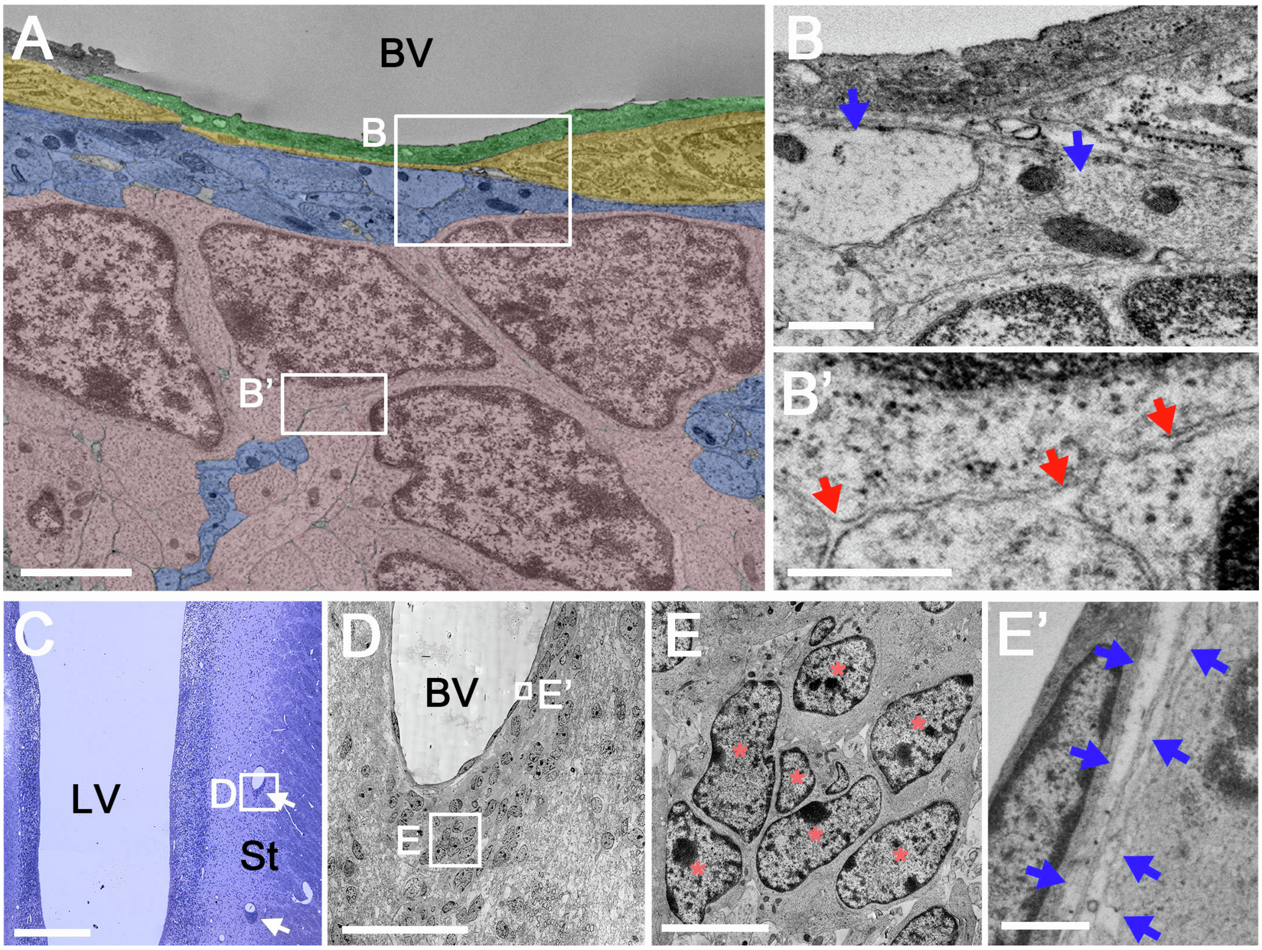

Figure S3. Ultrastructural analysis of neuroblast-blood vessel and neuroblast-neuroblast interaction, related to Figure 3.

A-B': A large neuroblast cluster located in tier 3 of the dorsolateral V-SVZ. The cluster is closely associated with vasculature (A, pink: neuroblasts, blue: astrocytes, green: endothelium, orange: pericytes). Higher magnification views of the boxed areas in A are shown in B and B'. Astrocytic endfeet (blue arrows) are interposed between the blood vessel and the adjacent neuroblasts (B). Neuroblasts are densely packed, forming narrow intercellular spaces (B', red arrows).

C-E': Neuroblast clusters in the striatum. A semi-thin toluidine blue-stained section (C) shows large neuroblast clusters (white arrows) surrounding blood vessels in the striatal parenchyma. A low magnification TEM image of the boxed area in C is shown in (D). Higher magnification views of the boxed areas in D are shown in E and E'. Neuroblasts are marked with pink asterisks (E). Thin astrocytic endfeet between the blood vessel and neuroblasts are indicated with blue arrows (E').

Abbreviations: LV, lateral ventricle; St, striatum; BV, blood vessel. Scale bars, 500 μm: C; 50 μm: D; 5 μm: E; 2 μm: A; 1 μm: E'; 0.5 μm: B, B'.

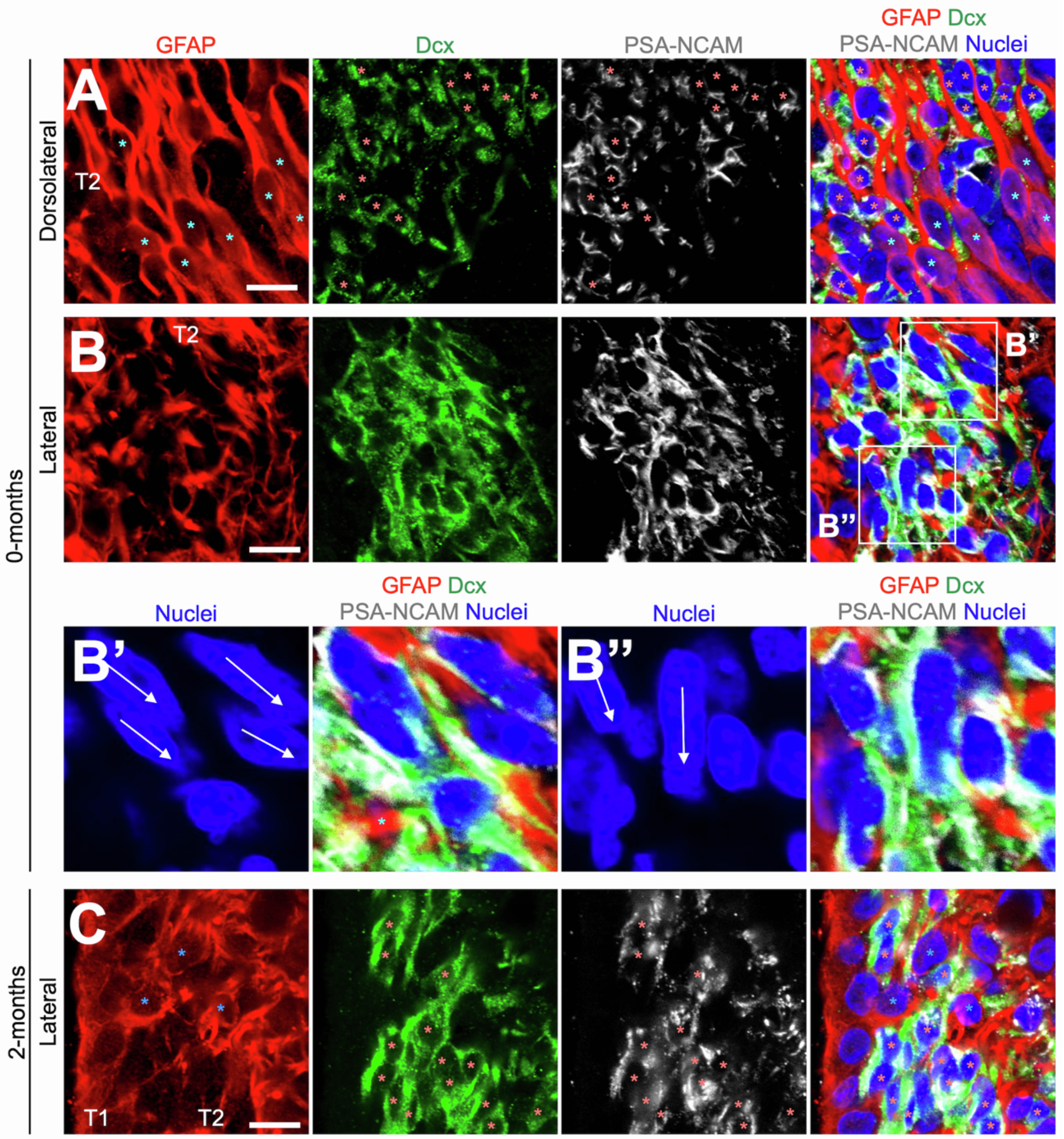

Figure S4. Identification of cell types in tier 2 of 0-month-old and 2-month-old microminipigs, related to Figures 3 and 4.

A-C: Representative images of the coronal V-SVZ sections from 0-month-old (A-B'') and 2-month-old (C) microminipigs stained for Dcx (green), GFAP (red), and PSA-NCAM (white). Nuclei were stained with Hoechst 33342 (blue). Light blue (A), pink (A, C), and dark blue (C) asterisks indicate GFAP+ radial glia, Dcx+PSA-NCAM+ neuroblasts, and GFAP+ astrocytes, respectively. Arrows (B' and B'') indicate cellular orientation. Boxed areas in (B) are shown at higher magnification in (B') and (B'').

T1, tier 1; T2, tier 2. Scale bars, 10 μm.

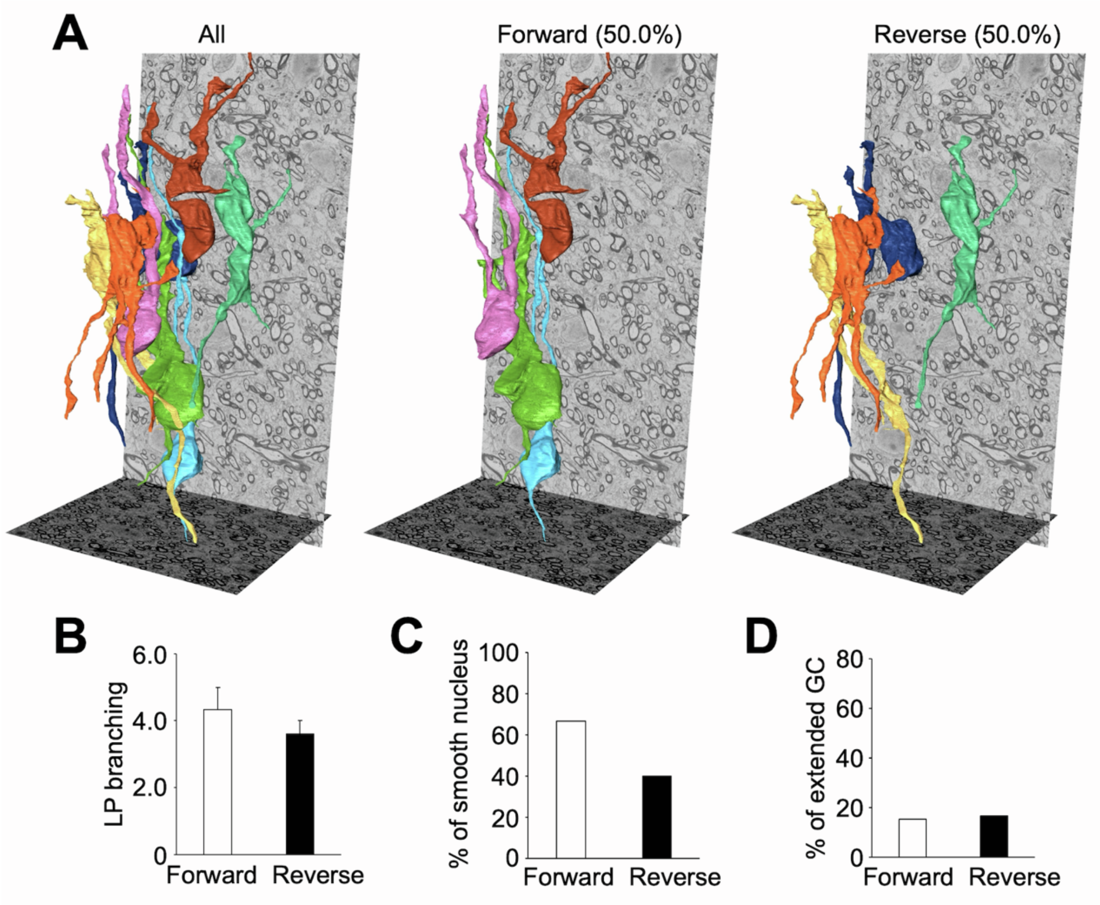

Figure S5. Three-dimensional morphology of neuroblast clusters located farther from blood vessels in tier 3

A: Representative SBF-SEM images of cluster of migratory neuroblasts located farther from blood vessels in tier 3 of the V-SVZ in 2-month-old microminipigs. The directions of neuroblasts are defined based on the directionality of blood vessel-associated neuroblasts shown in Figure 7A.

B: Number of leading process branches in forward- and reverse-migrating neuroblasts.

C: Proportion of cells with a smooth nucleus in forward- and reverse-migrating neuroblasts.

D: Proportion of cells with an extended growth cone in forward- and reverse-migrating neuroblasts.

Table S1. Ultrastructural characteristics of cell types in the postnatal V-SVZ of microminipigs.

	Neuroblasts	Radial glial cells	Astrocytes	Ependymal cells
**Location**	Tiers 1-3	0 months: tier 1/tier 2 (inner)<br>2 months: (-)	0 months: tiers 1/2 (few)<br>tier 3 (many)<br>2 months: tiers 1-3	Ventricular surface
**Contour**	Smooth	Elongated, Smooth	Irregular	Frequent adherens junctions<br>Interdigitated
**Cytoplasm**	Scant<br>Free ribosomes<br>Microtubules	Intermediate filaments<br>9+0 cilium	Light<br>Intermediate filaments<br>9+0 cilium	Basal bodies<br>Multiple 9+2 cilia<br>Apical mitochondria
**Nucleus**	Elongated, Small	Irregular<br>Occasionally invaginated	Irregular, Electron lucent<br>Occasionally invaginated	Spherical, Large

Supplementary Methods

Immunohistochemistry

Immunohistochemistry was performed as described previously with slight modification (Akter et al., 2020). Briefly, the microminipigs were deeply anesthetized by intramuscular injection of a mixture of 0.04 mg/kg medetomidine, 5 mg/kg ketamine hydrochloride, and 0.2 mg/kg midazolam or by inhalation of isoflurane, and then fixed by transcardiac perfusion with 4% paraformaldehyde (PFA) in 0.1 M phosphate buffer (PB) (pH 7.4). The brains were postfixed in the same fixative at 4 ºC. 70-µm-thick coronal sections were cut using a vibratome (VT-1200S, Leica). Coronal sections (70 µm-thick) from the olfactory bulb (OB) to anterior V-SVZ regions were incubated for 40 min at RT in blocking solution (10% normal donkey serum and 0.4% TritonX-100 in PBS), overnight at 4 ºC with the primary antibodies, and 3 h at RT with AlexaFluor-conjugated secondary antibodies (1:1,000, Invitrogen) in the blocking solution. The following primary antibodies were used: goat anti-doublecortin (Dcx) (1:500, Santa Cruz Biotechnology); mouse anti-glial fibrillary acidic protein (GFAP) monoclonal (1:500, Sigma). Nuclei were stained with Hoechst 33342 (1:5,000, Sigma).

Transmission Electron Microscopy

Neonatal and 2-month-old microminipig brains were fixed by transcardiac perfusion with 2.5% glutaraldehyde (GA) and 2% PFA in 0.1 M PB (pH 7.4) and postfixed in the same fixative at 4 ºC. Three hundred-µm-thick coronal sections from OB to anterior V-SVZ regions were cut using a vibratome (VT-1200S, Leica), followed by treatment with 2% OsO_4 in the same buffer for 2 h at 4°C and treatment with 1% uranyl acetate/50% ethanol for 1 h at 4°C. The sections were then dehydrated in a graded ethanol series, placed in propylene oxide, and embedded in Durcupan resin. Semi-thin sections (2.0 µm-thick) were sequentially cut using an ultramicrotome (UC6, Leica Microsystems) with a diamond knife (histo, DiATOME). The sections were stained with 1% toluidine blue solution, and those of interest were chosen under a light microscope CX23 (Olympus). Serial ultra-thin sections (60-70 nm-thick) were cut from the embedded semi-thin sections using an ultramicrotome (UC6, Leica Microsystems) with a diamond knife (SYM2045, SYNTEK), and then stained with 2% uranyl acetate in distilled water for 15 min and with modified Sato's lead solution for 5 min. Sections were examined using a transmission electron microscope (JEM-1400Plus; JEOL, Tokyo, Japan). Images at 8,000× magnification were stitched using Shot Meister software (TEMography.com) (Figure 3B-D, F, G, J-L, N-Q, W-Y', Figure 4B, D-G', J-L', Q).

For quantification of neuroblast contact with surrounding cells (Figures 3, 4), whole-cell images were acquired at a magnification of 8,000×. Only membrane segments

in direct contact with other cells or structures were analyzed. The segments were classified according to the contacting element—radial glia, neuroblasts, astrocytes, blood vessels, other cells, or no contact—and their length was measured using Fiji software (National Institutes of Health, USA). The percentage of membrane length for each category relative to the total measured membrane was then calculated.

Serial Block-Face Scanning Electron Microscopy

Two-month-old microminipig brains were fixed by transcardiac perfusion with 2.5% GA and 2% PFA in 0.1 M PB (pH 7.4) and postfixed in the same fixative at 4 ºC. The V-SVZ tissue blocks dissected from the 300-μm-thick coronal sections were treated with 2% OsO_4 and 1.5% potassium ferricyanide in PBS for 1 h at 4°C, 1% thiocarbohydrazine for 20 min at RT, 2% aqueous OsO_4 for 30 min at RT, and lead aspartate solution for 30 min at 65°C. The samples were then dehydrated in a graded ethanol series, treated with dehydrated acetone, and embedded in Durcupan resin containing Ketjenblack powder.

Serial image sequences were acquired at a resolution of 6 nm/pixel and at 80 nm steps. Sequential images were processed using FIJI. Orientation of each neuroblast was determined by the direction of the leading process and the position of the centrosome. Growth cone extension and collapse are defined as a distal membrane expansion that is more than three times larger or smaller, respectively, than the diameter of the leading process.

Statistics

The Shapiro-Wilk test was used to assess normality and equal variances between groups. When data did not meet the assumptions of normality or equal variance, the Steel-Dwass test or Kruskal-Wallis test was applied. For comparisons between two independent groups in the SBF-SEM analyses (Figure 7), Fisher’s exact test or an unpaired *t*-test was used.
