# Supplementary material for: Cytoarchitecture of neurogenic niche and neuroblast clusters in the postnatal microminipig brain
Source: Stem Cell Reports. 2026 Apr 16;21(5):102893. doi: 10.1016/j.stemcr.2026.102893 (PMC13163217; doi:10.1016/j.stemcr.2026.102893)
Supplement: Document S2. Article plus supplemental information [file mmc2.pdf]

STEM CELL REPORTS
Article
ISSCR
OPEN ACCESS

Cytoarchitecture of neurogenic niche and neuroblast clusters in the postnatal microminipig brain

Daijiro Kojima,[1,2,13] Masato Sawada,[1,3,13] Taisei Ishimaru,[4] Nodoka Ito,[4] Shinichiro Tateyama,[1,2] Kazuhide Adachi,[2] Hiroaki Kawaguchi,[5,6] Noriaki Satake,[7] Vicente Herranz-Pérez,[8,9] José Manuel García-Verdugo,[8,12] Yuichi Hirose,[2] Nobuhiko Ohno,[10,11] Naoko Kaneko,[1,4,*] and Kazunobu Sawamoto[1,3,14,*]

[1]Department of Developmental and Regenerative Neurobiology, Institute of Brain Science, Nagoya City University Graduate School of Medical Sciences, Nagoya 467-8601, Japan
[2]Department of Neurosurgery, Fujita Health University, Toyoake 470-1192, Japan
[3]Division of Neural Development and Regeneration, National Institute for Physiological Sciences, Okazaki 444-8585, Japan
[4]Laboratory of Neuronal Regeneration, Graduate School of Brain Science, Doshisha University, Kyotanabe 610-0394, Japan
[5]Laboratory of Veterinary Pathology, Kitasato University School of Veterinary Medicine, Towada 034-8628, Japan
[6]Laboratory of Veterinary Histopathology, Joint Faculty of Veterinary Medicine, Kagoshima University, Kagoshima 890-0065, Japan
[7]Fuji Micra Inc., Fujinomiya 418-0005, Japan
[8]Laboratory of Comparative Neurobiology, Cavanilles Institute, University of Valencia, CIBERNED–ISCIII, 46980 Valencia, Spain
[9]Department of Cell Biology, Functional Biology and Physical Anthropology, University of Valencia, CIBERNED–ISCIII, 46100 Burjassot, Spain
[10]Department of Anatomy, Division of Histology and Cell Biology, Jichi Medical University, School of Medicine, Shimotsuke 329-0498, Japan
[11]Division of Ultrastructural Research, National Institute for Physiological Sciences, Okazaki 444-8585, Japan
[12]Deceased
[13]These authors contributed equally
[14]Lead contact
*Correspondence: nkaneko@mail.doshisha.ac.jp (N.K.), sawamoto@med.nagoya-cu.ac.jp (K.S.)
https://doi.org/10.1016/j.stemcr.2026.102893

SUMMARY

The ventricular-subventricular zone (V-SVZ) is the largest neurogenic niche in the postnatal mammalian brain, but its organization and migratory dynamics remain poorly understood in gyrencephalic species. Here, we provide ultrastructural and three-dimensional characterization of the V-SVZ neuroblasts in postnatal microminipigs, the smallest pig strain with unique advantages for experimental neuroscience. Transmission electron microscopy revealed developmental changes in cell composition and cytoarchitecture, with migratory neuroblasts consistently associated with glial cells and vasculatures. Notably, serial block-face scanning electron microscopy revealed that tier 3 neuroblasts, a gyrencephalic-specific population, formed elongated, chain-like clusters aligned along vessels, with conserved intracellular features such as polarized organelle distributions and growth cone extension. Radial glial fibers were prominent in neonates but diminished with age, suggesting a developmental shift to vascular scaffolds as primary migration guides. These findings establish microminipigs as a tractable gyrencephalic model for studying postnatal neurogenesis, offering new opportunities for translational research on brain repair.

INTRODUCTION

The postnatal mammalian brain retains a remarkable capacity to generate new neurons. In rodents, radial glial cells, the embryonic neural stem cells, rapidly differentiate into ependymal cells and astrocytes after birth in most brain regions, whereas those in the ventricular-subventricular zone (V-SVZ) along the lateral walls of the lateral ventricles give rise to postnatal neural stem cells that continue to generate immature neurons (Chaker et al., 2024). These newborn neurons, or neuroblasts, form elongated chain-like aggregates and migrate toward their destinations under physiological and pathological conditions (Bressan and Saghatelyan, 2020; Nakajima et al., 2021). In humans, active neurogenesis occurs during the neonatal period but rapidly declines within the first 6 months of life (Sanai et al., 2004; 2011). Neuroblasts migrate not only to the OB but also to the prefrontal and entorhinal cortices (Kim et al., 2025; Nascimento et al., 2024; Paredes et al., 2016; Sanai et al., 2011). The neonatal human V-SVZ is divided into three tiers: tier 1, facing the ventricles and densely packed with cells; tier 2, containing dispersed neuroblasts; and tier 3, adjacent to the corpus callosum and containing clusters of neuroblasts (Paredes et al., 2016). Tiers 2 and 3, regions characteristic of gyrencephalic brains (Kim et al., 2025), are regarded as containing clusters of neuroblasts migrating toward their final destinations. However, the three-dimensional morphology of these clusters (e.g., spherical versus elongated chain-like structures) and their spatial relationships with surrounding tissues, which may underlie their directional migration, remain unclear.

Large animals such as primates and pigs have been used as preclinical models to investigate postnatal neurogenesis and neuronal migration in the human brain. Macaque monkeys, Old World monkeys with human-like gyrencephalic brain structures, have been used extensively (Gil-Perotin et al., 2009; Kornack and Rakic, 2001; Pencea

Published by Elsevier Inc. on behalf of International Society for Stem Cell Research.
This is an open access article under the CC BY license (http://creativecommons.org/licenses/by/4.0/).

et al., 2001), but their use is increasingly constrained by ethical considerations. Common marmosets, small New World monkeys, are advantageous due to their manageable size and shared cytoarchitectural features with the human V-SVZ; however, their brains are lissencephalic (Akter et al., 2020; Sawamoto et al., 2011). Pigs, by contrast, possess gyrencephalic brains, and postnatal V-SVZ neurogenesis as well as its alterations under pathological conditions have been reported (Alonso-Alconada et al., 2024; Jinnou et al., 2025; Morton et al., 2017; Porter et al., 2022; Torrijos-Saiz et al., 2025).

Nevertheless, conventional pigs are large (about 1.4 kg at birth and 100–300 kg as adults), making experimental interventions challenging. Minipigs, such as the Gottingen minipig (about 0.5 kg at birth and 35–45 kg as adults), have been developed to address this limitation (Ayuso et al., 2020). However, investigating postnatal neurogenesis requires tracking V-SVZ-derived neuroblasts across various brain regions and developmental stages, including adulthood, which would benefit from an even smaller gyrencephalic model.

Here, we report V-SVZ neurogenesis in the microminipig (*Sus scrofa domesticus*), a unique miniature pig strain derived from potbelly pigs and recognized as the smallest pig model (about 0.3 kg at birth and 25 kg as adults) (Sugiyama et al., 2011; Takasu et al., 2015). Transmission electron microscopy (TEM) revealed developmental changes in the cellular composition of the postnatal microminipig V-SVZ and structural similarities to the primate V-SVZ. Furthermore, serial block-face scanning electron microscopy (SBF-SEM) allowed us to reconstruct the 3D morphology of individual neuroblasts and their chain-like clusters in tier 3. These observations provide insights into potential migration routes of neuroblasts from the V-SVZ to parenchymal regions in gyrencephalic brains during postnatal development.

RESULTS

Developmental changes in cellular morphology and distribution in the postnatal microminipig V-SVZ

Microminipigs, recognized as the smallest pig model (Sugiyama et al., 2011; Takasu et al., 2015) (Figure 1A), typically reach sexual maturity between 3 and 5 months of age and have a lifespan of 12–18 years. We examined brains from neonatal (0-month-old), juvenile (2-month-old), and adult (3-year-old) microminipigs (Figures 1B–1D and 2A–2C) and identified the major gyri and sulci as previously reported in minipigs (Bjarkam et al., 2017; Yun et al., 2011) (Figures 1B–1D and 2A–2C). Well-developed OBs were visible in ventral and sagittal views (Figures 1C, 1D, 2B, and 2C), and coronal sections showed lateral ventricles extending into the OB (Figure 1B′), similar to those in minipigs (Bjarkam et al., 2017). These observations indicate that microminipig brains are smaller but retain the structural characteristics of conventional pig brains (Bjarkam et al., 2017; Yun et al., 2011).

To assess postnatal development in the V-SVZ and OB, we stained sections from neonatal, 2-month-old, and adult animals for doublecortin (Dcx, neuroblasts), polysialylated neural cell adhesion molecule (PSA-NCAM, neuroblasts), and glial fibrillary acidic protein (GFAP, astrocytes) (Figures 1E–1J, 2D–2Q, and S1A–S1H). Similar to the cytoarchitecture of neonatal pig and human brains (Kim et al., 2025; Paredes et al., 2016; Porter et al., 2022), the dorsal V-SVZ consisted of three layers: tier 1, a dense layer of Dcx+PSA-NCAM+ neuroblasts adjacent to the lateral ventricles; tier 2, containing dispersed Dcx+PSA-NCAM+ neuroblasts; and tier 3, containing clusters of Dcx+PSA-NCAM+ neuroblasts adjacent to the corpus callosum (Figure S1G). Hereafter, we mainly used an anti-Dcx antibody to visualize neuroblasts in the microminipig V-SVZ.

In tier 1, at 0 months, many Dcx+ neuroblasts were distributed up to 150 μm from the ventricular surface (Figures 1E and 1F) and were closely associated with GFAP+ radial fibers. By 2 months, tier 1 had thinned, with fewer Dcx+ neuroblasts, and radial fibers were replaced by meshwork-like GFAP+ fibers (Figures 1H and 1I). Similar age-dependent changes were also observed in the core of the OB (Figures 2F and S1). In adults, despite reduced thickness, Dcx+ cells persisted in the astrocytic fiber meshwork, particularly in the dorsomedial V-SVZ across rostrocaudal levels (Figures 2D, 2E, and 2G). To examine proliferative activity in neuroblasts in the adult V-SVZ, brain sections were immunostained for the proliferation marker Ki67 in addition to Dcx. Very few Ki67+ cells weakly expressing Dcx were detected in tier 1 (Figure S2A), and no such cells were observed in tier 2 or 3, indicating that proliferative neuroblasts are rare in the adult microminipig V-SVZ. Nevertheless, Dcx+ cells were continuously distributed throughout the microminipig V-SVZ even in adulthood, in contrast to human brains.

In tiers 2 and 3, large Dcx+ clusters were observed at birth, some associated with putative blood vessels surrounded by GFAP+ processes (Figures 1G and G′), as reported previously in neonatal pig and human brains (Paredes et al., 2016; Porter et al., 2022). By 2 months, these clusters had decreased in number, and their connections had become looser (Figure 1J), with GFAP+ processes extending between neuroblasts, suggesting that they had separated into smaller clusters.

In adults, Dcx+ cell clusters were still observed in tiers 2 and 3 and in the corpus callosum (Figures 2H–2K), in contrast to human brains (Paredes et al., 2016). GFAP+ processes were extensively interposed between neuroblasts

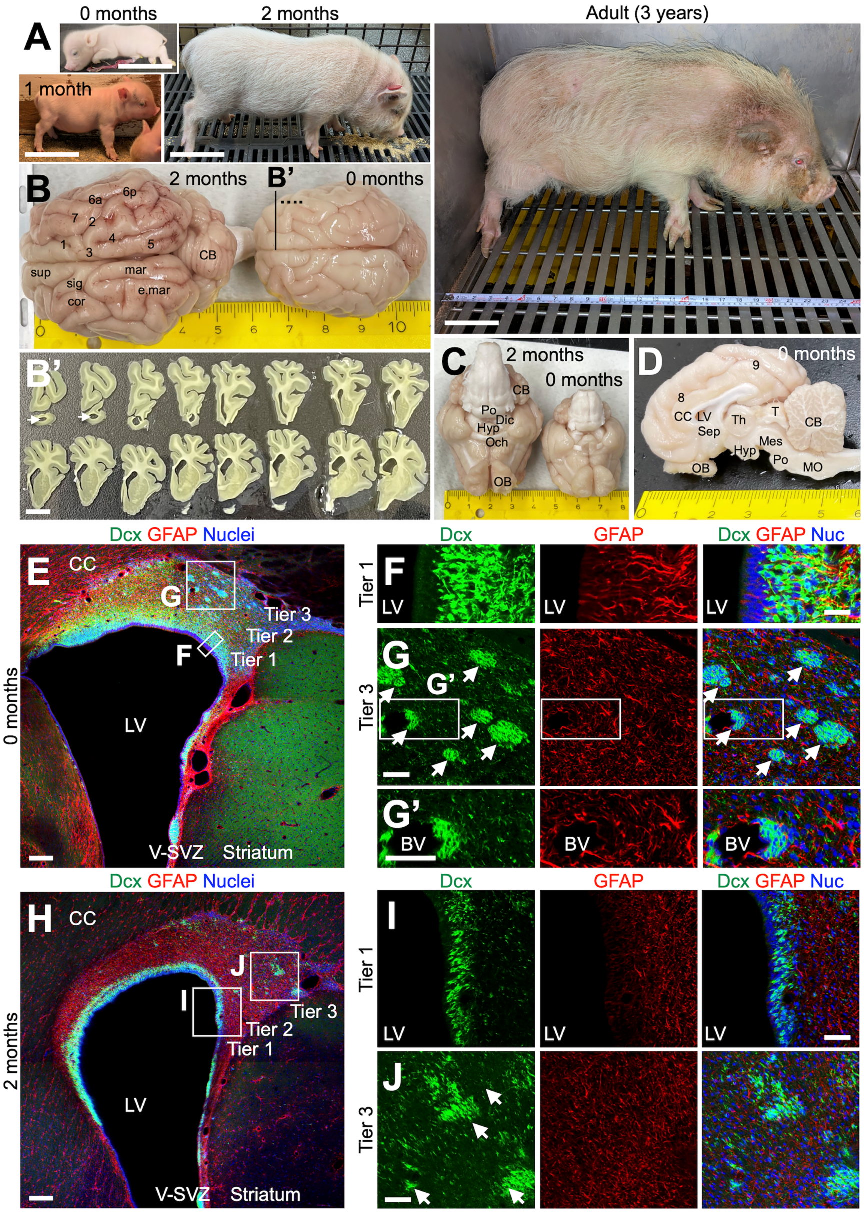

Figure 1. Brain structure and V-SVZ cytoarchitecture of postnatal microminipigs

(A) Representative images of microminipigs at 0, 1, and 2 months and at the adult stage (3 years).

(B–D) Representative images of the brain surface structure of 0-month-old and 2-month-old microminipigs. Dorsal (B), ventral (C), and sagittal (D) views of the microminipig brains are shown. Numbers in (B and D) indicate major sulci: 1, cruciate sulcus; 2, coronal sulcus; 3, ansate sulcus; 4, lateral sulcus; 5, ectolateral sulcus; 6a, anterior suprasylvii sulcus; 6p, posterior suprasylvii sulcus; 7, sulcus naris; 8, cingulate sulcus; 9, splenial sulcus. sup, superior frontal gyrus; sig, sigmoid gyrus; cor, coronal gyrus; mar, marginal gyrus; e.mar, ectomarginal gyrus. OB, olfactory bulb; Och, optic chiasm; Hyp, hypothalamus; Dic, diencephalon; CC, corpus callosum; LV, lateral ventricle; Sep, septum; Th, thalamus; T, tectum; Mes, mesencephalon; Po, pons; MO, medulla oblongata; CB, cerebellum. Representative images of coronal brain sections in 0-month-old microminipigs are shown in (B′). Arrows (B′) indicate lateral ventricles extending into the OB.

(E–J) Representative images of the coronal V-SVZ sections in 0-month-old (E–G′) and 2-month-old (H–J) microminipigs stained for Dcx (green) and GFAP (red). Nuclei were stained with Hoechst 33342 (blue). Boxed areas in (E) and (H) are enlarged in (F and G) and (I and J), respectively. A hole wrapped with GFAP+ processes in (G′) is a putative blood vessel. Arrows (G and J) indicate clusters of Dcx+ neuroblasts in tier 3. LV, lateral ventricle; V-SVZ, ventricular-subventricular zone; CC, corpus callosum. Scale bars: 10 cm in (A); 1 cm in (B′); 200 μm in (E and H); 50 μm in (F, G, G′, I, and J).

rather than surrounding the clusters, and direct neuroblast-neuroblast contacts were occasionally observed, suggesting age-dependent changes in neuroblast and astrocyte interactions. While some Dcx+ cells co-express oligodendrocyte lineage marker Olig2 in the postnatal neocortex in common marmosets (Akter et al., 2020), Dcx+ cells in the adult microminipig brain were co-labeled with the migrating neuroblast marker PSA-NCAM (Figures 2N and 2O) but not with GFAP, Olig2, or the microglial marker Iba1 (Figures 2G–2M and S2B–S2E′), supporting the possibility that these cells were neuroblasts. PSA-NCAM immunoreactivity in neuroblasts was significantly lower in the corpus callosum than in tier 2 and the OB (Figures 2P, 2Q, and S2F), suggesting region-dependent migratory activity. Together, these findings indicate that neuroblast migration persists in the adult microminipig brain, although the mode of migration differs from that observed during postnatal development.

Ultrastructural architecture of tiers 1 and 2 in the microminipig V-SVZ at birth

To investigate the postnatal development of V-SVZ cytoarchitecture and neuroblast migration at the ultrastructural level, we performed TEM analysis. Cell-type

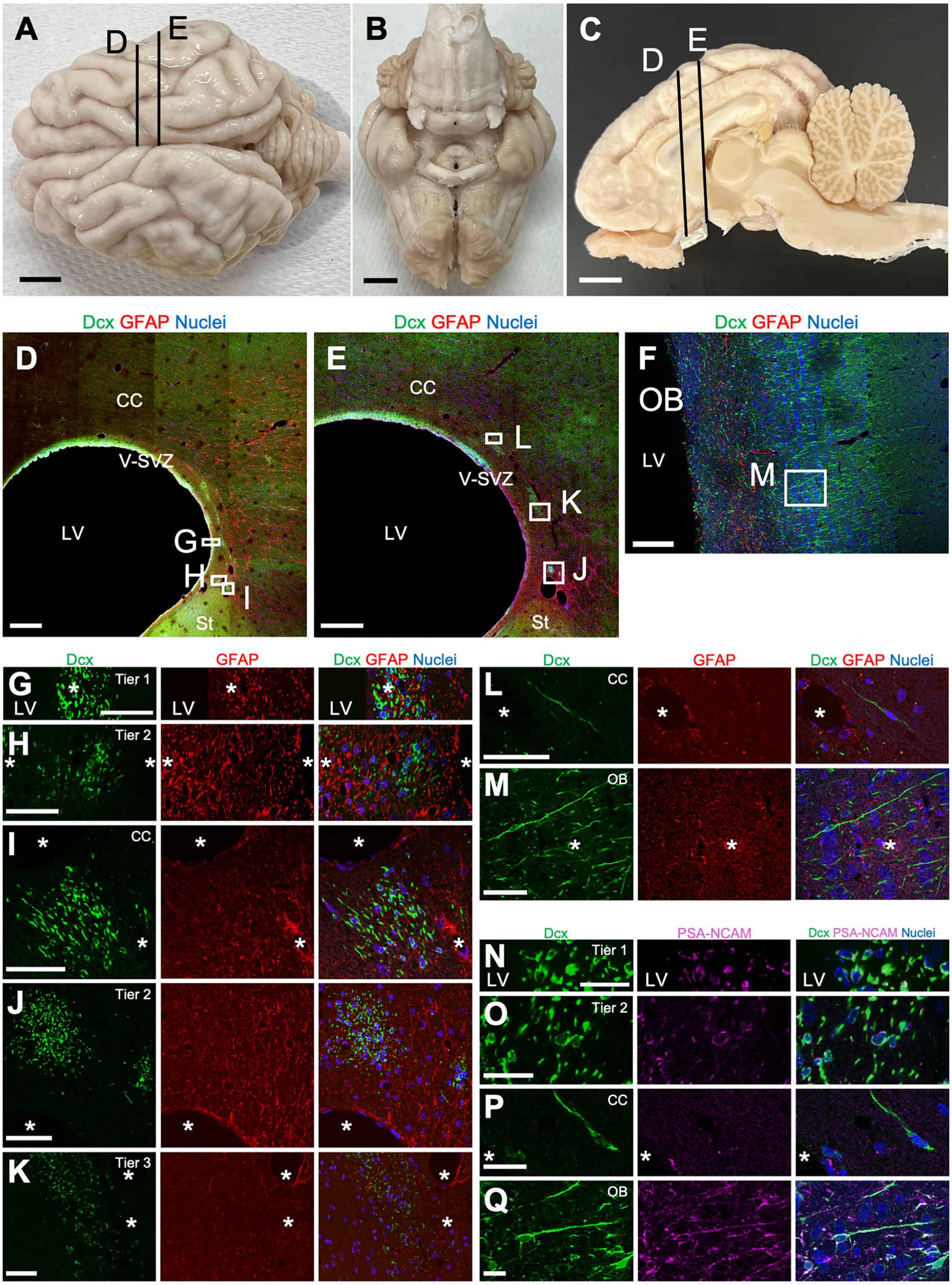

Figure 2. Brain structure and V-SVZ cytoarchitecture of an adult microminipig

(A–C) Representative images of the brain surface structure of a 36-month-old microminipig. Dorsal (A), ventral (B), and sagittal (C) views are shown. Numbers (A and C) indicate major sulci: 1, cruciate sulcus; 2, coronal sulcus; 3, ansate sulcus; 4, lateral sulcus; 5, entolateral

(legend continued on next page)

classification followed established criteria (Akter et al., 2020; Doetsch et al., 1997; Torrijos-Saiz et al., 2025) (Table S1). At 0 months (Figures 3A–3Y′ and S3), a thick, high-density layer up to 300 μm from the ventricular surface (tiers 1 and 2) was observed in the dorsal wall (Figures 3A–3L). Numerous extracellular gaps were present throughout this layer (Figures 3C–3F, 3G, and 3J), unlike neighboring cortical (Figures 3V–3Y′) and striatal regions (Figures S3C–S3E′). Tier 1 (30–40 μm thick; Figure 3B, T1) consisted mainly of ependymal cells with multiple motile cilia along the ventricular surface (Figures 3C, 3D, and 3E′) and radial glial cells. Most radial glial bodies were above ependymal cells, but some contacted the ventricle (Figure 3C) and bore primary cilia (Figures 3D and 3E). Sparse neuroblasts, with small elongated nuclei, smooth contours, and numerous microtubules, similar to those in rodent and primate brains (Akter et al., 2020; Doetsch et al., 1997), were interspersed among radial glia (Figure 3C). In the inner region of tier 2 (40–100 μm from ventricle), radial glial cell bodies formed small clusters (Figures 3F and S4A), consistent with features of outer radial glia. In the outer two-thirds, dense neuroblasts and radial glial processes predominated (Figures 3B and 3G–3L). Over 60% of neuroblasts here formed small clusters of 2–6 individuals (Figures 3G and 3J) with intercellular free spaces (Figure 3I, red arrows), typical of migratory neuroblasts in the V-SVZ and RMS (Doetsch et al., 1997).

To explore potential migration routes, we analyzed neuroblast contacts with putative scaffold structures and their morphological polarity. Neuroblast clusters often contacted radial glial fibers (Figures 3H, 3I′, 3I″, and 3R). Neuroblasts with more than 20% contact showed significantly larger radial migration angles than those with less than 20% (Figure 3S), suggesting that radial glia serve as scaffolds for radial migration. Few neuroblasts were associated with blood vessels (Figures 3J and 3T), separated from the vascular basal lamina by a thin layer of astrocytic processes (Figures 3K and 3L). Our data suggest that contact with blood vessels has less influence on migration orientation during the neonatal stage compared with radial glial processes (Figure 3U). These findings suggest that in the dorsolateral wall, neuroblasts mainly migrate radially along radial glial processes toward the neocortex.

In tier 2 of the lateral wall (Figures 3M–3Q), some small neuroblast clusters remained associated with radial glial processes (Figures 3N, 3O, 3P, S4B, and S4B′), whereas many neuroblasts exhibited elongated nuclei and microtubule-rich processes oriented parallel to the ventricular surface. These cells were frequently aligned with neighboring neuroblasts, consistent with tangential migration (Figures 3O′, 3Q, and S4B–S4B″). Quantification showed that neuroblasts with close contact with radial glia had significantly larger radial orientation angles than those with less contact, even in the lateral wall, although angles were significantly smaller than those in the dorsolateral wall (Figure 3S). Vascular association was rare and did not affect orientation (Figures 3T and 3U). Taken together, in the lateral wall, neuroblasts predominantly migrate tangentially along the ventricle, with radial glial contact still modulating orientation.

Ultrastructure of neuroblast clusters in tier 3 of the microminipig V-SVZ at birth

In contrast to tiers 1 and 2, tier 3, extending toward the white matter, was densely populated with neurons, glial cells, and their processes, although myelin sheaths were still sparse and thin at this age (Figures 3V–3Y′). Consistent with previous reports in human infants and piglets (Kim et al., 2025; Paredes et al., 2016; Porter et al., 2022; Torrijos-Saiz et al., 2025), several large clusters, occasionally containing more than 100 neuroblasts, were observed (Figures 3V and 3W). Neuroblasts within these clusters were tightly packed with minimal intercellular spaces (Figures 3X–3Y′ and S3A–S3B′). To assess the spatial extent of these clusters, we examined their morphology in serial semi-thin sections spanning >50 μm rostrocaudally. All 16 identified clusters were organized around cross-sections of small blood vessels ensheathed by astrocytic endfeet (Figures S3B and S3E′), extending across multiple sections. This organization is consistent with neuroblast migration

sulcus; 6a, anterior suprasylvii sulcus; 6p, posterior suprasylvii sulcus; 7, sulcus naris; 8, cingulate sulcus; 9, splenial sulcus. sup, superior frontal gyrus; sig, sigmoid gyrus; cor, coronal gyrus; mar, marginal gyrus; e.mar, ectomarginal gyrus.

(D–M) Confocal images of the coronal brain sections at different rostrocaudal levels (indicated in A and/or C) immunostained for Dcx (green) and GFAP (red). Nuclei were stained with Hoechst 33342 (blue). Boxed areas around the LV (D and E) and the OB (F) are enlarged in (G–L) and (M), respectively. Putative blood vessels (tubular structures wrapped with GFAP+ processes in (G–M) are indicated with asterisks.

(N–Q) Confocal images of the V-SVZ (N and O), CC (P), and OB (Q) immunostained for Dcx and PSA-NCAM. Nuclei were stained with Hoechst 33342.

OB, olfactory bulb; Och, optic chiasm; Hyp, hypothalamus; Dic, diencephalon; CC, corpus callosum; LV, lateral ventricle; Sep, septum; Th, thalamus; T, tectum; Mes, mesencephalon; Po, pons; MO, medulla oblongata; CB, cerebellum; V-SVZ, ventricular-subventricular zone. Asterisks: blood vessels.

Scale bars: 10 mm in (A–C); 500 μm in (D and E); 200 μm in (F); 50 μm in (G–M); 20 μm in (N–Q).

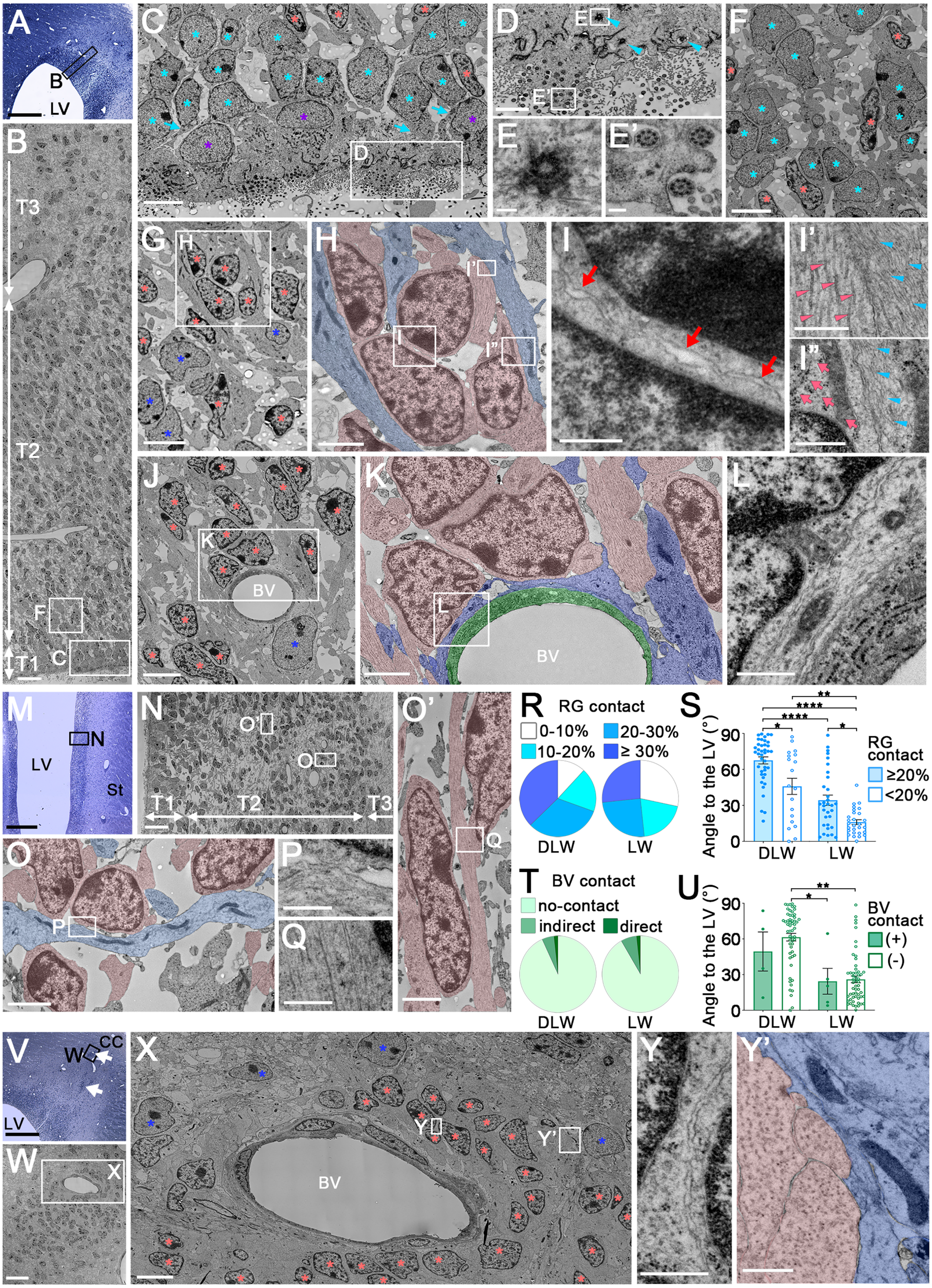

Figure 3. TEM of the V-SVZ in 0-month-old microminipigs

(A–L) Tiers 1–2 in the dorsolateral wall (DLW). Toluidine blue-stained semi-thin section (A) and low magnification TEM (B). In tier 1 (C–E), ependymal cells with 9 + 2 cilia (E′), radial glial (RG) extending apical processes (C, cyan arrows), single neuroblasts, and 9 + 0 primary cilia

(legend continued on next page)

along the rostrocaudal axis using vascular scaffolds. Similar clusters were also seen in the medial striatum adjacent to the lateral V-SVZ (Figures S3C–S3E′). Astrocytes and their extensive processes surrounded the clusters (Figures 3X and 3Y′), but only a few thin astrocytic protrusions were within clusters (Figure S3A), resembling astrocytic tunnels in the RMS (Lois et al., 1996).

Taken together, these observations indicate that neonatal neuroblasts show region-specific modes of migration. Near the ventricle, a loose tissue with many spaces, they form small clusters migrating radially along radial glial processes or tangentially along each other. In contrast, in dense parenchyma with few spaces, they aggregate into large clusters migrating along blood vessels ensheathed by astrocytic endfeet. This suggests that the microenvironment, including radial glia or vasculature, shapes distinct migratory behaviors depending on location.

Ultrastructural architecture of the V-SVZ in 2-month-old microminipigs

By 2 months, tiers 1 and 2 had shrunk to less than 80 μm in thickness in both the dorsolateral wall (Figures 4A–4H) and the lateral walls (Figures 4I–4L′ and S4C). Among ependymal cells lining the ventricle, type B1 astrocytes with an apical protrusion bearing a primary cilium (Figures 4L and 4L′) were rarely observed. Radially oriented glial nuclei and processes were absent; instead, astrocytes with irregular contours and light cytoplasm containing intermediate filaments were distributed (Figure 4D). Neuroblasts typically formed small clusters of 2–5 cells (Figures 4D–4G). While average cluster size was similar between the 0- and 2-month groups (Figure 4M), the percentage of plasma membranes contacting other neuroblasts was increased significantly in the latter group (Figure 4N). Despite these contacts, intercellular free spaces indicative of active migration remained visible between membranes (Figure 4L′). Individual and clustered neuroblasts were extensively associated with astrocytic cell bodies/processes (Figures 4E, 4L′, 4O, and S4C).

To examine the effect of the age-dependent changes in V-SVZ composition on neuroblast migration direction, we compared their orientation relative to the ventricular wall (Figure 4P). In the 2-month dorsolateral wall, orientation was diverse, with only 10% of nuclei oriented radially (>60°) toward the corpus callosum. The mean angle was significantly smaller than at birth, when about 64% were oriented radially. In the lateral wall, the largely parallel orientation was similar between ages; however, radially oriented neuroblasts observed at birth were absent at 2 months. Thus, within 2 months, radial migration toward adjacent parenchyma markedly decreased, coinciding with the loss of radial glia.

In tier 3 regions, many axons with thick myelin sheaths were present, and the boundary with the corpus callosum was indistinct in the lateral wall (Figure 4Q). Large tightly packed clusters seen in neonates were absent; neuroblasts appeared in small clusters or individually (Figures 4F–4H and 4Q–4S′). These cells were surrounded by astrocytic processes, and occasionally contacted blood vessels via astrocytic endfeet (Figures 4G and 4R) or directly with myelinated axons (Figure 4G, 4H, 4R, and 4S). Even in dense parenchyma, intercellular free spaces between neuroblasts were still evident (Figure 4S′), suggesting that migration in

(D, arrowheads; E) were observed. RGs were also present in the inner one-third of tier 2 (F). In outer tier 2 (G–L), small neuroblast clusters (H, pink) contacted RG processes (H, cyan). Neuroblasts showed narrow intercellular spaces (I, red arrows), microtubule-rich processes (I′, pink arrowheads), and scant cytoplasm with many free ribosomes (I″, pink arrows). RG processes contained intermediate filaments (I′ and I″, cyan arrowheads). A neuroblast cluster (J and K, pink) contacted a blood vessel encased by astrocytic processes (K, green: endothelium, blue: astrocytes). Higher magnification images are from boxed areas in B (C and F), C (D), D (E and E′), G (H), H (I–I″), J (K), and K (L).
(M–Q) Tiers 1 and 2 in the lateral wall (LW). Semi-thin section (M) and low-magnification TEM (N) show the tiered structure. In tier 2, neuroblasts (O and O′, pink) contacted RG processes (O, cyan) or aligned along other neuroblast processes (O′, pink). Higher magnification images are from boxed areas in M (N), N (O and O′), O (P), and O′ (Q).
(R) Percentage of neuroblast membrane in contact with RG (DLW: $n = 59$ cells, LW: $n = 60$ cells).
(S) Nuclear axis angle relative to the ventricular surface, grouped by RG contact (≥20% vs. <20%) (DLW, ≥20%: $n = 40$ cells, <20%: $n = 19$ cells; LW, ≥20%: $n = 32$ cells, <20%: $n = 28$ cells, Steel-Dwass test). Bar graph indicates mean ± SEM.
(T) Percentage of neuroblast membrane contacting blood vessels (DLW: $n = 59$ cells, LW: $n = 60$ cells).
(U) Nuclear axis angle with (+) or without (−) vascular contact (DLW, +: $n = 4$ cells, −: $n = 55$ cells; LW, +: $n = 5$ cells, −: $n = 55$ cells, Steel-Dwass test). Bar graph indicates mean ± SEM.
(V–Y′) Large neuroblast clusters with blood vessels in tier 3. Semi-thin section (V, arrows) and TEM (W–Y′) show densely packed neuroblasts with minimal intercellular spaces (Y), surrounded by astrocytic processes (Y′, blue). Higher magnifications from boxed areas in V (W), W (X), and X (Y and Y′).
Color-coded asterisks: purple, ependymal cells; cyan, radial glia; pink, neuroblasts; blue, astrocytes. LV, lateral ventricle; St, striatum; BV, blood vessel; CC, corpus callosum; T1, tier 1; T2, tier 2; T3, tier 3. $*p < 0.05$, $**p < 0.01$, $***p < 0.001$, $****p < 0.0001$. Scale bars, 500 μm in (A, M, and V); 20 μm in (B, N, and W); 5 μm in (C, F, G, J, and X); 2 μm in (D, H, K, O, and O′); 1 μm in (L and Y′); 0.5 μm in (I, I′, I″, P, Q, and Y); 0.2 μm in (E and E′).

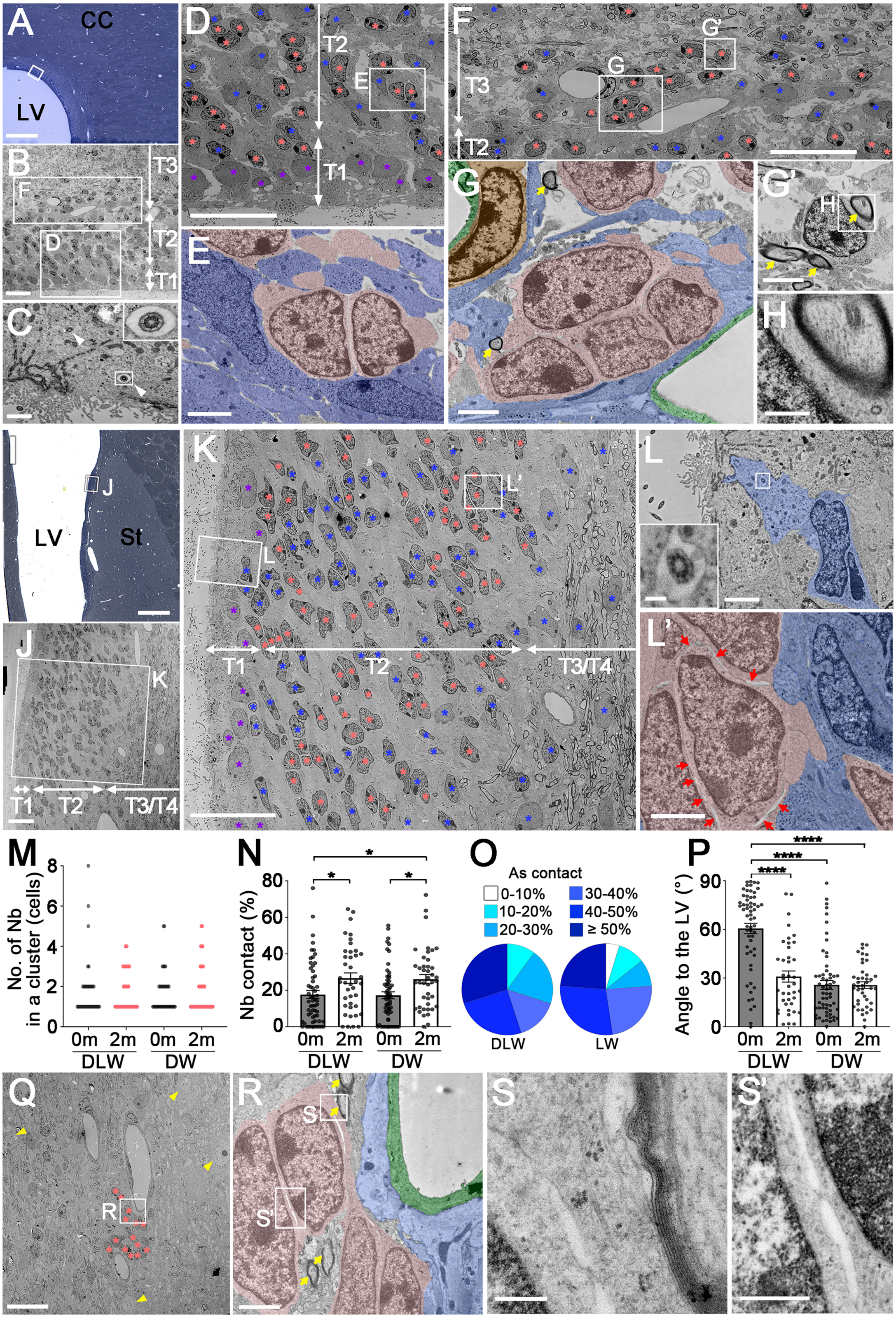

(legend on next page)

parenchymal regions (corpus callosum and striatum) was reduced but still observed along blood vessels and axons.

Three-dimensional morphology of individual neuroblasts in tier 3

Clusters of Dcx+ neuroblasts in tier 3 are a unique feature of gyrencephalic animals, including pigs and humans (Kim et al., 2025; Paredes et al., 2016; Porter et al., 2022; Torrijos-Saiz et al., 2025), but whether these cells show typical migratory morphology remains unclear. To address this issue, we imaged more than 2,000 consecutive 80-nm-thick sections from 2-month-old microminipigs using SBF-SEM. In blood vessel-associated neuroblast clusters, neuroblasts showed unipolar or bipolar smooth cell contours, dark cytoplasm, and a small Golgi apparatus (Figures 5A and 5B), consistent with features reported in mice (Matsumoto et al., 2019).

Among clustered neuroblasts, 56.7% extended an unbranched leading process resembling that in mice; the rest of the cells had one (23.3%), two (13.3%), or three (6.7%) branches in their leading process (Figures 5B and 5C). Growth cones, either extended or collapsed, were seen at the tip (Nakajima et al., 2024) (Figure 5B, brown). Mitochondria, Golgi apparatus, and paired centrioles accumulated in the proximal leading process or in a swelling, as in mice (Matsumoto et al., 2019) (Figure 5A; yellow arrows, asterisks, and white arrows, respectively). These results indicate that tier 3 neuroblasts in postnatal microminipigs retain typical migratory morphology.

Three-dimensional morphology of neuroblast clusters in tier 3

The ultrastructural morphology of cluster-forming neuroblasts in tier 3 was analyzed using serial SBF-SEM images. As seen in immunostaining and TEM, some neuroblasts within clusters contacted blood vessels via thin astrocytic processes containing intermediate filaments (Figures 6A, 6C, and 6C′). These clusters also interacted with both myelinated and non-myelinated axons (Figures 6A and 6C). Intercellular free spaces between neuroblasts (Figures 4R and 4S′) were consistently observed (Figures 6A and 6B′), supporting the idea that these cells migrate actively within the cluster.

To determine overall cluster shape, we reconstructed clusters' 3D morphology (Figure 6D). Clusters were not spherical aggregates but elongated chain-like structures composed of neuroblasts with migratory morphology (Figure 6D). Because such clusters were frequently associated with blood vessels (Figures 1 and 4), we next examined the spatial relationship between them. Neuroblasts were partially in contact with vessel walls (Figures 6C, 6C′, and 6D′) and aligned parallel to them (Figure 6D), suggesting that blood vessels may serve as migratory scaffolds, similar to blood vessel-associated clusters observed in the same region at birth (Figures 3V–3X).

Figure 4. TEM of the V-SVZ in 2-month-old microminipigs

(A–H) Tiers 1–3 in the dorsolateral wall (DLW). Toluidine blue-stained semi-thin section (A) and low-magnification TEM (B) show the tiered structure. In tier 1 (C and D), 9 + 0 primary cilia (C, arrowheads, inset) were rare among multiciliated ependymal cells (D). Tier 2 (D and E) contained many astrocytes and neuroblasts (single cells or small clusters) in close association (E, pink: neuroblasts, blue: astrocytes). In tier 3 (F–H), a neuroblast cluster contacted blood vessels (G, pink: neuroblasts, green: endothelium, orange: pericyte, blue: astrocytes). Neuroblasts frequently contacted myelinated axons (G–G′, yellow arrows; H). Higher magnification images are from boxed areas in B (F and D), D (E), F (G and G′), and G′ (H).

(I–L′) Tiers 1–4 in the lateral wall (LW). Semi-thin section (I) and low-magnification TEM (J) show the tiered structure. In tier 1, ependymal cells lined the ventricular surface (K), and a type B1 astrocyte with an apical protrusion and 9 + 0 primary cilium was observed (L, inset). Astrocytes (single or clustered) were distributed throughout tiers (K) and closely associated with neuroblasts (L′, blue, astrocytes; pink, neuroblasts; red arrows: intercellular spaces). Higher magnification images are from boxed areas in I (J), J (K), and K (L and L′).

(M) Dot plots showing the number of neuroblasts per cluster at 0 months (0 m) and 2 months (2 m) (DLW, 0 m: $n = 68$ cells, 2 m: $n = 72$ cells; LW, 0 m: $n = 63$ cells, 2 m: $n = 72$ cells).

(N) Percentage of neuroblast membrane in contact with other neuroblasts at 0 m and 2 m (DLW, 0 m: $n = 59$ cells, 2 m: $n = 40$ cells; LW, 0 m: $n = 60$ cells, 2 m: $n = 42$ cells, Steel-Dwass test). Bar graph indicates mean ± SEM.

(O) Percentage of neuroblast membrane in contact with astrocytes at 2 m (DLW: $n = 40$ cells; LW: $n = 42$ cells).

(P) Nuclear axis angle relative to the ventricular surface at 0 m and 2 m (DLW, 0 m: $n = 59$ cells, 2 m: $n = 40$ cells; LW, 0 m: $n = 60$ cells, 2 m: $n = 41$ cells, Steel-Dwass test). Bar graph indicates mean ± SEM.

(Q–S′) Neuroblast clusters in the striatum. Low magnification image (Q) shows a cluster surrounded by myelinated axons and striatal neurons (yellow arrowheads). Neuroblasts contacted myelinated axons and astrocytic endfeet on blood vessels (R, pink: neuroblasts, green: endothelium, blue: astrocytes, yellow arrows: myelinated axons). Higher magnification images are from boxed areas in Q (R) and R (S and S′).

Color-coded asterisks: purple, ependymal cells; pink, neuroblasts; blue, astrocytes. LV, lateral ventricle; St, striatum; CC, corpus callosum; T1, tier1; T2, tier2; T3, tier3. $*p < 0.05$, $****p < 0.0001$. Scale bars, 500 μm in (A and I); 20 μm in (B, D, F, J, K, and Q); 2 μm in (E, G, G′, L, L′, and R); 1 μm in (C); 0.5 μm in (H and S′); 0.2 μm in (L) inset, (S).

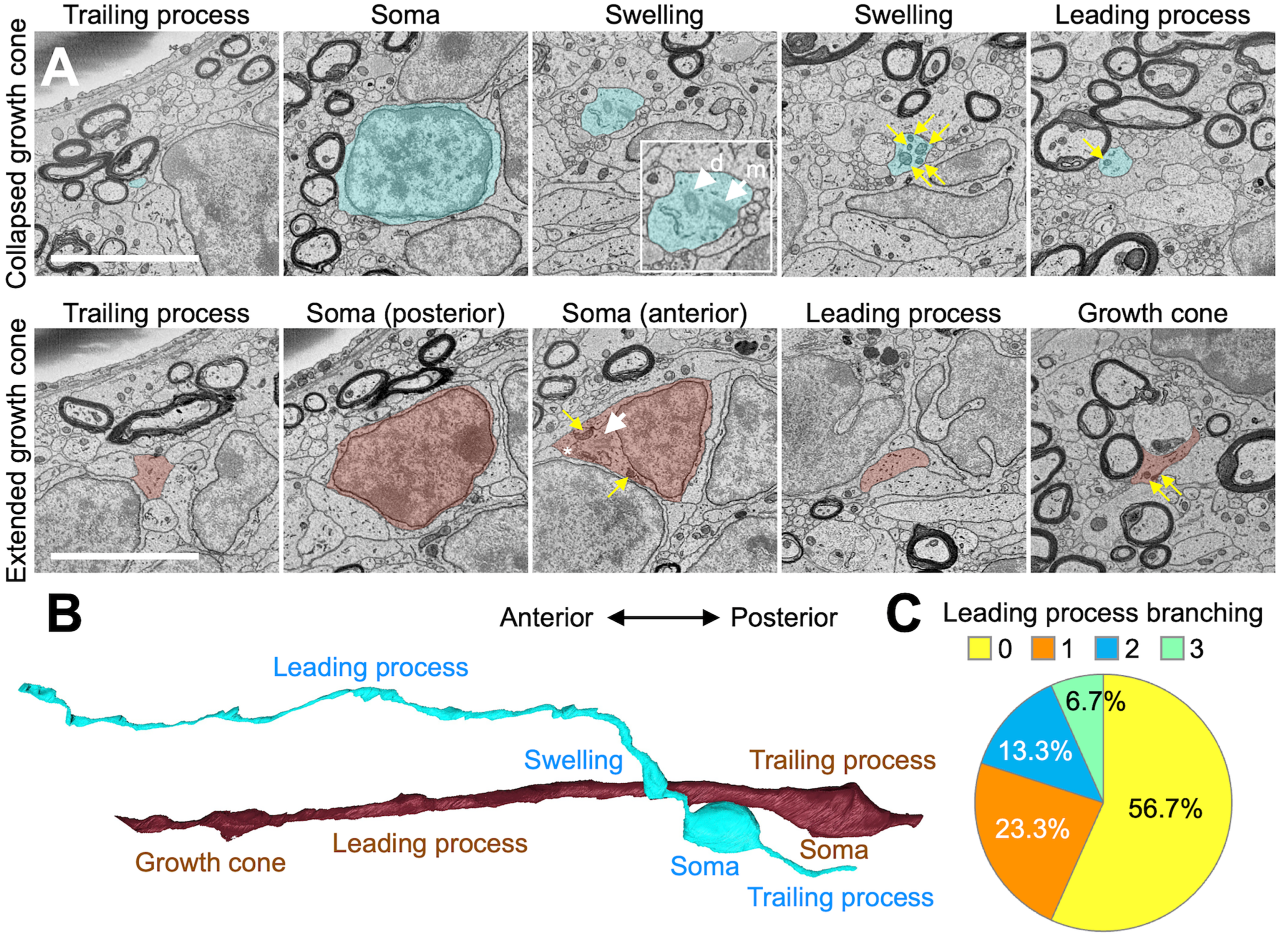

Figure 5. Three-dimensional morphology of individual neuroblasts in blood vessel-associated clusters in tier 3

(A) Representative SBF-SEM images of migratory neuroblasts with (bottom) or without (top) the growth cone in tier 3 of the V-SVZ in 2-month-old microminipigs. The migratory neuroblasts consist of the trailing process, soma, swelling (occasional), leading process, and the growth cone. White and yellow arrows indicate centrosomes and mitochondria, respectively. Asterisk [bottom, soma (anterior)] indicates the Golgi apparatus. Swelling in the neuroblast with a collapsed growth cone (top, swelling) is magnified in the right bottom box (m, mother centriole; d, daughter centriole).

(B) Representative 3D reconstructions of migratory neuroblasts shown in (A).

(C) Proportion of neuroblasts with unbranched and branched leading processes.

Scale bars, 10 μm.

To assess the orientation of neuroblasts within clusters, we determined the direction of the leading process in neuroblasts based on the centriole position. Quantitative analysis showed that 70.0% (21/30) of neuroblasts extended their leading process forward and 30.0% (9/30) in the reverse direction (Figure 7A), indicating directional migration. We then examined how migration direction related to the leading process and nuclear morphology. Forward-migrating neuroblasts had fewer branches in the leading process and a higher proportion of smooth nuclei than reverse-migrating cells (Figures 7B and 7C), suggesting more efficient leading process extension and subsequent somal translocation.

We further analyzed growth cone morphology in relation to migration direction. Similar to chain-forming neuroblasts in mice, microminipig neuroblasts extended their growth cone along neighboring cells in the cluster (Figures 7D–7D″). Extended growth cones were significantly more frequent in forward-migrating neuroblasts than in reverse-migrating ones (Figure 7E), suggesting active extension of the leading process during migration.

Finally, to investigate the relationship between blood vessel association and neuroblast cluster morphology, we analyzed a cluster located farther from blood vessels in tier 3 (Figure S5). In contrast to blood vessel-associated clusters, this non-associated cluster consisted

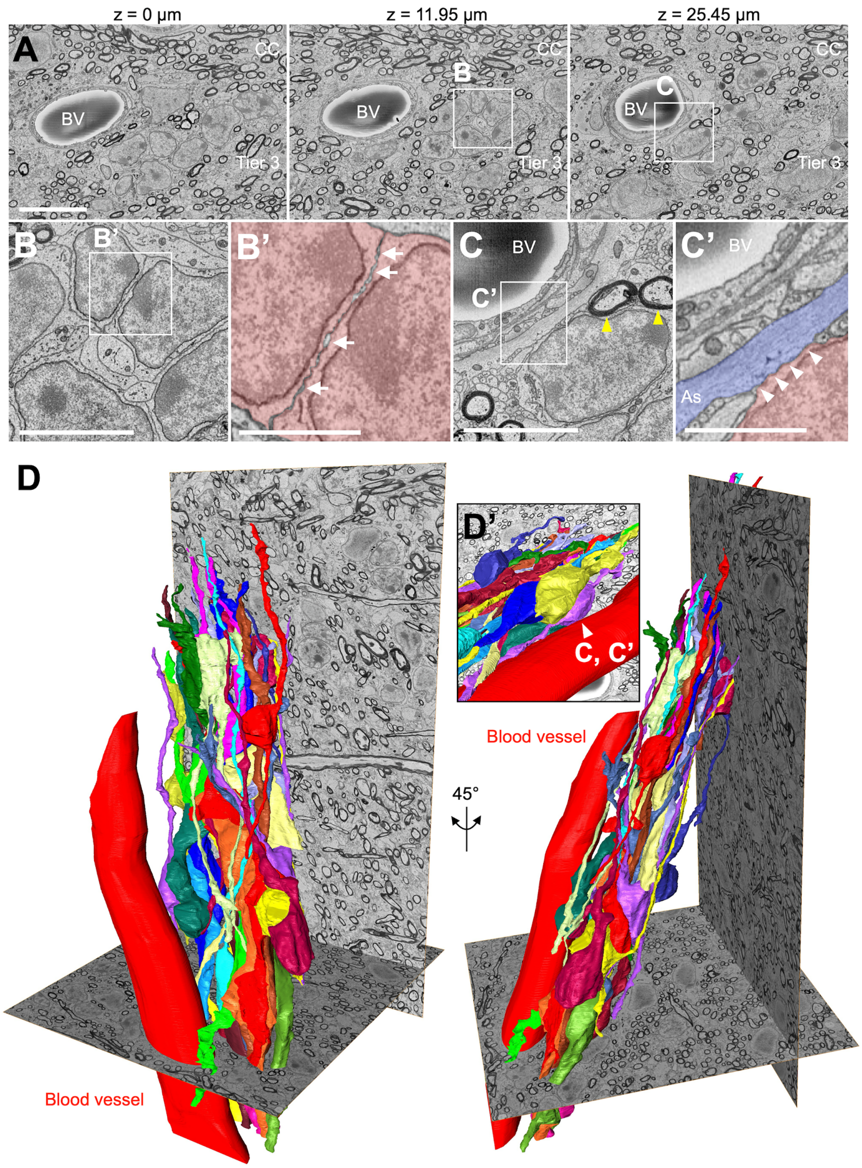

Figure 6. Three-dimensional morphology of blood vessel-associated neuroblast clusters in tier 3

(A–C′) Representative SBF-SEM images of blood vessel-associated clusters of migratory neuroblasts in tier 3 of the V-SVZ in 2-month-old microminipigs. Boxed areas in (A) are magnified in (B, C). Boxed areas in (B) and (C) are further magnified in (B′) and (C′), respectively. Arrows (B′) indicate intercellular free spaces at the contact site of two neuroblasts. Yellow and white arrowheads (C and C′) indicate neuroblast contact to myelinated axons and an astrocytic process (As), respectively. BV, blood vessel.

(D) Three-dimensional reconstructions of clusters of migratory neuroblasts and an associated blood vessel shown in (A–C′). Contact site between neuroblast and blood vessel (C and C′) is shown in (D′). Scale bars: 10 µm in (A); 5 µm in (B and C); 2 µm in (B′ and C′).

of neuroblasts with irregular morphology and reduced organization (Figure S5A). At the single-cell level, neuroblasts in this cluster did not exhibit a clear relationship between migratory directionality and nuclear morphology (Figure S5C) and showed increased branching of leading processes together with reduced growth cone extension (Figures S5B and S5D). Together, these results suggest that blood vessels contribute to the directional migration of neuroblasts in chain-like clusters in tier 3.

Taken together, these results suggest that tier 3 neuroblasts form chain-like elongated clusters and migrate actively along blood vessels in postnatal microminipigs.

DISCUSSION

In this study, we revealed developmental changes in cell composition and cytoarchitecture of the V-SVZ cells at the ultrastructural level in postnatal microminipigs. Migratory neuroblasts were consistently associated with surrounding glial cells and blood vessels. Three-dimensional reconstruction showed that cluster-forming neuroblasts in tier 3, a population characteristic of gyrencephalic brains (Kim et al., 2025; Paredes et al., 2016), retained typical migratory morphology and assembled into elongated, chain-like aggregates aligned along blood vessels. These findings indicate that the microenvironment

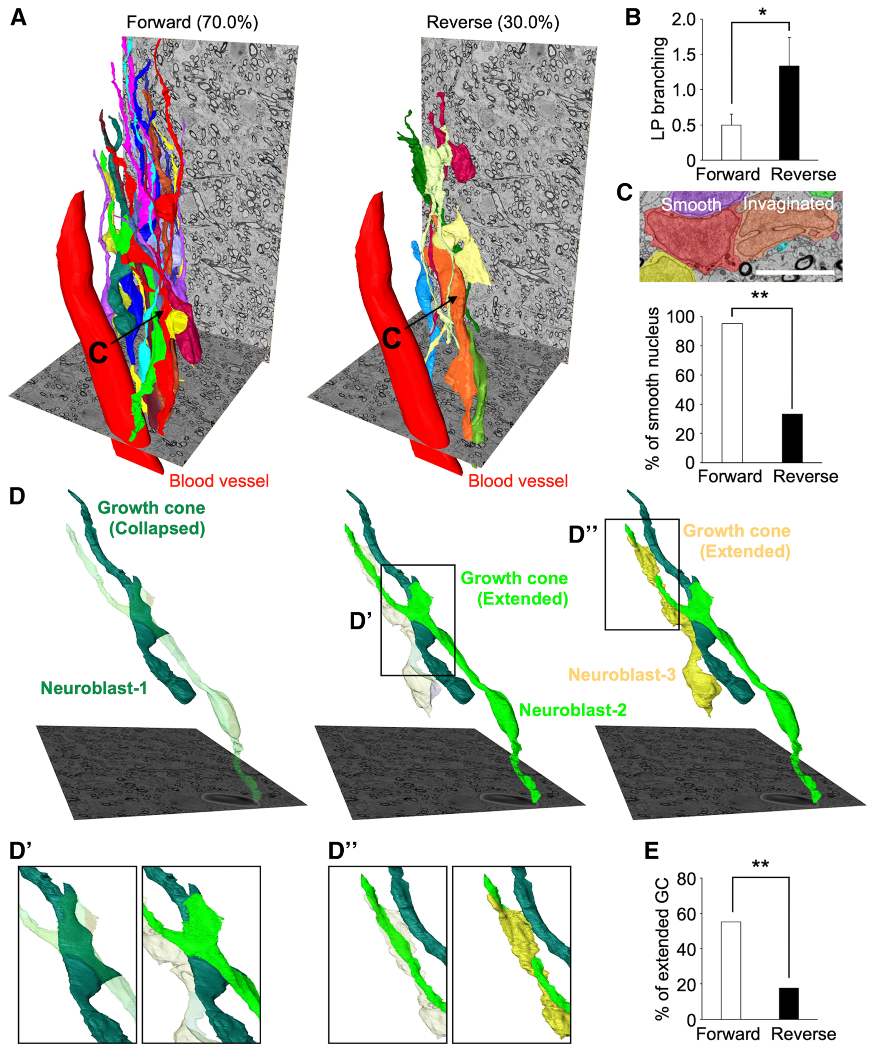

Figure 7. Morphology of growth cone, leading process, and nucleus in cluster-forming neuroblasts in tier 3

(A) Three-dimensional reconstructions of forward- and reverse-migrating neuroblasts. Nuclear morphology of the neuroblasts indicated by arrows is shown in (C).

(B) Number of leading process branching in forward- and reverse-migrating neuroblasts (unpaired t test). Bar graph indicates mean ± SEM.

(C) Proportion of cells with smooth nucleus in forward- and reverse-migrating neuroblasts (Fisher's exact test). Representative SBF-SEM image of cells with smooth (red) and invaginated (orange) nucleus, whose 3D morphology is shown in (A), is also shown.

(D) Representative 3D reconstruction images of cluster-forming neuroblasts. Growth cones of neuroblast-2 and -3 are enlarged in (D′) and (D″), respectively.

(E) Proportion of cells with extended growth cone in forward- and reverse-migrating neuroblasts (Fisher's exact test). Scale bars, 5 μm. $^{*}p < 0.05$, $^{**}p < 0.01$.

provides region- and age-specific scaffolds that shape neuroblast migration patterns.

Our SBF-SEM analysis further demonstrated that microminipig neuroblasts displayed conserved intracellular features associated with migration. In mice, the Golgi apparatus, centrosomes, and mitochondria accumulate in the proximal domain of the leading process or its swelling, where they regulate cytoskeletal remodeling and energy metabolism (Bressan et al., 2020; Matsumoto et al., 2019). Neuroblasts also extend a growth cone at the tip of their leading process, driving efficient movement (Nakajima et al., 2024). The observation of similar organelle distributions and growth cone morphology in microminipigs (Figure 5) suggests that common cellular machinery underlies migration across species.

Blood vessels are well established as migratory scaffolds for neuroblasts in the postnatal brain. In rodents, they guide neuroblasts along the V-SVZ-RMS-OB pathway, the corpus callosum, and toward lesion sites after stroke (Nakajima et al., 2021). In common marmosets, blood vessels also support neuroblast migration toward the cerebral cortex during postnatal development (Akter et al., 2020). In microminipigs, we observed that neuroblasts were attached to vessel walls and oriented parallel to them (Figure 6), supporting the idea that vascular scaffolds and their molecular milieu create a favorable environment for directed migration in the postnatal gyrencephalic brain.

While vascular scaffolds are used throughout life, radial glia serve as migratory guides primarily during embryonic and early postnatal periods. In mice, radial glia disappear soon after birth, but can persist or reappear following brain injury to direct neuroblasts toward the injured neocortex (Jinnou et al., 2018). In neonatal microminipigs, radially oriented neuroblasts were frequently observed in close association with radial glial fibers, suggesting that radial glia guide neuroblasts radially toward adjacent

parenchymal regions, including the corpus callosum, neocortex, and striatum, during early postnatal development (Figures 1 and 3). In contrast, blood vessels appeared to preferentially guide neuroblasts along the rostrocaudal axis (Figures 3 and 6), a route that may direct cells toward the OB, which is more prominently developed in pigs than in primates. Taken together, these observations suggest that, in the postnatal brain, neuroblasts utilize either radial glial fibers or blood vessels depending on their migration direction. As radial glial fibers diminish during development, vascular scaffolds may become the predominant long-term guidance structure for neuroblast migration.

In higher mammals, including humans, outer radial glia are abundant during embryonic development and contribute to the expansion of the gyrencephalic cerebral cortex. In conventional pigs, neonatal outer radial glia become reactivated after injury, producing oligodendrocyte precursor cells that aid in functional recovery (Jinnou et al., 2025). Although further studies are required to clarify their role in microminipigs, we identified a similar population in inner-tier 2 of the neonatal V-SVZ (Figure 3), implying that radial glia in gyrencephalic species may have dual roles, as migratory scaffolds and as progenitors, supporting both brain development and repair.

Gyrencephalic species exhibit an expanded V-SVZ, in which the number of neuroblasts decreases as brain size increases during postnatal development (Kim et al., 2025; Paredes et al., 2016; Porter et al., 2022). While microminipigs share these general features, they uniquely maintain neuroblast clusters in adulthood, a property more reminiscent of lissencephalic species. Thus, microminipigs may represent an intermediate evolutionary state in which gyrencephalic brain organization coexists with partial preservation of lissencephalic-like maintenance of neuroblasts in adulthood. Dcx+ neuroblast clusters persist into adulthood, suggesting that neuroblast migration may remain active in the adult microminipig brain; however, their destinations remain unknown. Because Dcx+ neuroblasts are thought not to be newly generated in other gyrencephalic species (Bonfanti et al., 2024; La Rosa et al., 2018; Piumatti et al., 2018), it is possible that a subset of Dcx+ clusters observed in adult microminipigs (Figure 2) similarly represents immature, but not newly generated, neuroblasts.

Non-human primates, such as common marmosets and macaques, have been invaluable for elucidating postnatal human neurogenesis (Akter et al., 2020; Gil-Perotin et al., 2009; Kornack and Rakic, 2001; Pencea et al., 2001; Sawamoto et al., 2011). Ferrets have also served as a useful model for studying cortical folding, owing to their suitability for genetic manipulation (Shinmyo et al., 2022; Wang et al., 2024). However, there is a growing need for additional gyrencephalic animal models suitable for experimental manipulation. Microminipigs (Abe et al., 2018; Sugiyama et al., 2011; Takasu et al., 2015; Takeishi et al., 2018), which are smaller than previously reported miniature pigs (Ayuso et al., 2020), offer advantages for husbandry and enable surgical and pharmacological studies with greater feasibility. Establishing models of brain injury such as ischemic stroke or traumatic brain injury in microminipigs will facilitate the evaluation of regenerative strategies with direct translational potential.

In summary, our ultrastructural and 3D analyses reveal that microminipig neuroblasts retain conserved migratory machinery and undergo a developmental shift from radial glial to vascular scaffolds. These features, together with their tractable size and gyrencephalic brain, position microminipigs as a powerful preclinical model for testing strategies that enhance endogenous neurogenesis and migration after brain injury.

METHODS

Animals

Neonatal microminipigs (*Sus scrofa domesticus*) (0–3 days old, referred to as 0 month old; 0.3–0.5 kg; n = 5) and juvenile microminipigs (2 months old; 5–6 kg; n = 5) were obtained from Fuji Micra Inc. (Shizuoka, Japan). An adult female microminipig (36 months old; 28 kg; n = 1) was maintained at Kagoshima University. The room was maintained in a laminar flow of filtered air at a temperature of 24 ± 3°C, a relative humidity of 50 ± 20%, and a 12-h light/dark cycle. The animal had free access to tap water and was provided a normal chow diet (NcD; Kodakara 73; Marubeni Nisshin Feed Inc., Tokyo, Japan) on a daily basis. Research was performed according to the Institutional Guidelines for Animal Experiments and in compliance with the Japanese Act on Welfare and Management of Animals (Act No. 105 and Notification No. 6).

TEM

Brains of neonatal and 2-month-old microminipigs were fixed by transcardiac perfusion with 2.5% glutaraldehyde (GA) and 2% PFA in 0.1 M PB (pH 7.4) and postfixed in the same fixative at 4°C. 300-μm-thick coronal sections from the OB to anterior V-SVZ regions were prepared for semi-thin (2 μm thick) and ultra-thin (60–70 nm thick) sectioning. Cell types were identified as previously described (Akter et al., 2020; Doetsch et al., 1997; Torrijos-Saiz et al., 2025). Further details are provided in the supplemental information.

SBF-SEM

SBF-SEM observation of tier 3 of the V-SVZ was performed using a Merlin scanning electron microscope (Carl Zeiss) equipped with a 3View in-chamber ultramicrotome system

(Gatan). Segmentation of the cell membrane was performed using Microscopy Image Browser (Belevich et al., 2016). Three-dimensional reconstruction was performed using Amira software (Maxnet Co., Ltd, Tokyo, Japan). Further details are provided in the supplemental information.

Statistics

All statistical analyses were two-tailed and conducted using EZR (Kanda, 2013) or GraphPad Prism version 10.5.0 (GraphPad Software, La Jolla, CA, USA). A p value < 0.05 was considered statistically significant. Bar graphs indicate mean ± SEM. Further details are provided in the supplemental information.

RESOURCE AVAILABILITY

Lead contact

Requests for further information and resources should be directed to and will be fulfilled by the lead contact, Kazunobu Sawamoto (sawamoto@med.nagoya-cu.ac.jp).

Materials availability

This study did not generate new unique reagents.

Data and code availability

Any information required to reanalyze the data reported in this paper is available from the lead contact upon request.

ACKNOWLEDGMENTS

This work is dedicated to the memory of Prof. José Manuel García-Verdugo, who passed away before the submission of this paper. We thank N. Hattori, A. Imai (National Institute of Physiological Sciences), M. Tanaka, S. Nakamura (Nagoya City University), and the Research Equipment Sharing Center at Nagoya City University for providing technical support (JPMXS0441500024) and Sawamoto laboratory members for discussions. This work was supported by research grants from Japan Agency for Medical Research and Development (AMED) (25ym0126807, 24gm1210007, and 21bm0704033h0003 [to K.S.]); Japan Society for the Promotion of Science (JSPS) KAKENHI (19H04757, 19H04785, 18KK0213, 20H05700, and JP22H04926 [to K.S.], 20H03565, 21H05106, 23H02579, and 23K27270, [to N.K.], 18K14823, 21K06395, and 24K09660 [to M.S.], and 20K09377 [to K.A.]); Bilateral Open Partnership Joint Research Projects (14544620 [to K.S.] and JPJSBP120229939 [to N.K.]); Core-to-Core program "Neurogenesis Research & Innovation Center (NeuRIC)" (JPJSCCA20230007 to K.S.); JST FOREST Program (JPMJFR2146 to N.K.); Grant-in-Aid for Research at Nagoya City University (to M.S. and K.S.); Grant-in-Aid for Promotion on Co-Creative Urban Development in Nagoya City University (2412145 to K.S.); Grant-in-Aid for Outstanding Research Group Support Program in Nagoya City University grant number 2401101 (to K.S.); Cooperative Study Programs of National Institute for Physiological Sciences (to K.S.); the Mizutani Foundation for Glycoscience (to K.S.); Valencian Council for Education, Culture, University and Employment (CIPROM/2023/053) (to J.M.G.-V. and V.H.-P.); and the Takeda Science Foundation (to M.S. and K.S.).

AUTHOR CONTRIBUTIONS

D.K., M.S., T.I., N.I., S.T., K.A., H.K., N.S., N.O., and N.K. conducted the experiments; D.K., M.S., V.H.-P., J.M.G.-V., Y.H., N.O., N.K., and K.S. analyzed the results; D.K., M.S., N.K., and K.S. wrote the manuscript.

DECLARATION OF INTERESTS

N.S. is the president of Fuji Micra Inc.

SUPPLEMENTAL INFORMATION

Supplemental information can be found online at https://doi.org/10.1016/j.stemcr.2026.102893.

Received: October 27, 2025
Revised: March 18, 2026
Accepted: March 18, 2026
Published: April 16, 2026

REFERENCES

Abe, M., Kawaguchi, H., Miura, N., Akioka, K., Ushikai, M., Oi, S., Yukawa, A., Yoshikawa, T., Izumi, H., and Horiuchi, M. (2018). Diurnal Variation of Melatonin Concentration in the Cerebrospinal Fluid of Unanesthetized Microminipig. In Vivo (Athens, Greece) *32*, 583–590.

Akter, M., Kaneko, N., Herranz-Pérez, V., Nakamura, S., Oishi, H., García-Verdugo, J.M., and Sawamoto, K. (2020). Dynamic Changes in the Neurogenic Potential in the Ventricular–Subventricular Zone of Common Marmoset during Postnatal Brain Development. Cerebr. Cortex *30*, 4092–4109.

Alonso-Alconada, D., Gressens, P., Golay, X., and Robertson, N.J. (2024). Therapeutic hypothermia modulates the neurogenic response of the newborn piglet subventricular zone after hypoxia-ischemia. Pediatr. Res. *95*, 112–119.

Ayuso, M., Buyssens, L., Stroe, M., Valenzuela, A., Allegaert, K., Smits, A., Annaert, P., Mulder, A., Carpentier, S., Van Ginneken, C., and Van Cruchten, S. (2020). The Neonatal and Juvenile Pig in Pediatric Drug Discovery and Development. Pharmaceutics *13*, 44.

Belevich, I., Joensuu, M., Kumar, D., Vihinen, H., and Jokitalo, E. (2016). Microscopy Image Browser: A Platform for Segmentation and Analysis of Multidimensional Datasets. PLoS Biol. *14*, e1002340.

Bjarkam, C.R., Glud, A.N., Orlowski, D., Sørensen, J.C.H., and Palomero-Gallagher, N. (2017). The telencephalon of the Göttingen minipig, cytoarchitecture and cortical surface anatomy. Brain Struct. Funct. *222*, 2093–2114.

Bonfanti, L., La Rosa, C., Ghibaudi, M., and Sherwood, C.C. (2024). Adult neurogenesis and "immature" neurons in mammals: an evolutionary trade-off in plasticity? Brain Struct. Funct. *229*, 1775–1793.

Bressan, C., and Saghatelyan, A. (2020). Intrinsic Mechanisms Regulating Neuronal Migration in the Postnatal Brain. Front. Cell. Neurosci. *14*, 620379.

Chaker, Z., Makarouni, E., and Doetsch, F. (2024). The Organism as the Niche: Physiological States Crack the Code of Adult Neural Stem Cell Heterogeneity. Annu. Rev. Cell Dev. Biol. *40*, 381–406.

Doetsch, F., García-Verdugo, J.M., and Alvarez-Buylla, A. (1997). Cellular composition and three-dimensional organization of the subventricular germinal zone in the adult mammalian brain. J. Neurosci. *17*, 5046–5061.

Gil-Perotin, S., Duran-Moreno, M., Belzunegui, S., Luquin, M.R., and Garcia-Verdugo, J.M. (2009). Ultrastructure of the subventricular zone in Macaca fascicularis and evidence of a mouse-like migratory stream. J. Comp. Neurol. *514*, 533–554.

Jinnou, H., Rosko, L.M., Yamashita, S., Henmi, S., Prasad, J., Lam, V.K., Agaronyan, A., Tu, T.-W., Imamura, Y., Kuboyama, K., et al. (2025). Outer radial glia promotes white matter regeneration after neonatal brain injury. Cell Rep. Med. *6*, 101986.

Jinnou, H., Sawada, M., Kawase, K., Kaneko, N., Herranz-Pérez, V., Miyamoto, T., Kawaue, T., Miyata, T., Tabata, Y., Akaike, T., et al. (2018). Radial glial fibers support neuronal migration and regeneration after neonatal brain injury. Cell Stem Cell *22*, 128–137.e9.

Kanda, Y. (2013). Investigation of the freely available easy-to-use software "EZR" for medical statistics. Bone Marrow Transplant. *48*, 452–458.

Kim, J., Poddar, A., Sandoval, K., Chu, J., Horton, E., Cui, D., Nakamura, K., Lu, I.-L., Mui, M., Bartels, T., et al. (2025). An expanded subventricular zone supports postnatal cortical interneuron migration in gyrencephalic brains. Nat. Neurosci. *28*, 1598–1609.

Kornack, D.R., and Rakic, P. (2001). The generation, migration, and differentiation of olfactory neurons in the adult primate brain. Proc. Natl. Acad. Sci. USA *98*, 4752–4757.

La Rosa, C., Parolisi, R., Palazzo, O., Lévy, F., Meurisse, M., and Bonfanti, L. (2018). Clusters of DCX+ cells "trapped" in the subcortical white matter of early postnatal Cetartiodactyla (Tursiops truncatus, Stenella coeruloalba and Ovis aries). Brain Struct. Funct. *223*, 3613–3632.

Lois, C., García-Verdugo, J.M., and Alvarez-Buylla, A. (1996). Chain migration of neuronal precursors. Science *271*, 978–981.

Matsumoto, M., Sawada, M., García-González, D., Herranz-Pérez, V., Ogino, T., Bang Nguyen, H., Quynh Thai, T., Narita, K., Kumamoto, N., Ugawa, S., et al. (2019). Dynamic Changes in Ultrastructure of the Primary Cilium in Migrating Neuroblasts in the Postnatal Brain. J. Neurosci. *39*, 9967–9988.

Morton, P.D., Korotcova, L., Lewis, B.K., Bhuvanendran, S., Ramachandra, S.D., Zurakowski, D., Zhang, J., Mori, S., Frank, J.A., Jonas, R.A., et al. (2017). Abnormal neurogenesis and cortical growth in congenital heart disease. Sci. Transl. Med. *9*, eaah7029.

Nakajima, C., Sawada, M., and Sawamoto, K. (2021). Postnatal neuronal migration in health and disease. Curr. Opin. Neurobiol. *66*, 1–9.

Nakajima, C., Sawada, M., Umeda, E., Takagi, Y., Nakashima, N., Kuboyama, K., Kaneko, N., Yamamoto, S., Nakamura, H., Shimada, N., et al. (2024). Identification of the growth cone as a probe and driver of neuronal migration in the injured brain. Nat. Commun. *15*, 1877.

Nascimento, M.A., Biagiotti, S., Herranz-Pérez, V., Santiago, S., Bueno, R., Ye, C.J., Abel, T.J., Zhang, Z., Rubio-Moll, J.S., Kriegstein, A.R., et al. (2024). Protracted neuronal recruitment in the temporal lobes of young children. Nature *626*, 1056–1065.

Paredes, M.F., James, D., Gil-Perotin, S., Kim, H., Cotter, J.A., Ng, C., Sandoval, K., Rowitch, D.H., Xu, D., McQuillen, P.S., et al. (2016). Extensive migration of young neurons into the infant human frontal lobe. Science *354*, aaf7073.

Pencea, V., Bingaman, K.D., Freedman, L.J., and Luskin, M.B. (2001). Neurogenesis in the subventricular zone and rostral migratory stream of the neonatal and adult primate forebrain. Exp. Neurol. *172*, 1–16.

Piumatti, M., Palazzo, O., La Rosa, C., Crociara, P., Parolisi, R., Luzzati, F., Lévy, F., and Bonfanti, L. (2018). Non-Newly Generated, "Immature" Neurons in the Sheep Brain Are Not Restricted to Cerebral Cortex. J. Neurosci. *38*, 826–842.

Porter, D.D.L., Henry, S.N., Ahmed, S., Rizzo, A.L., Makhlouf, R., Gregg, C., and Morton, P.D. (2022). Neuroblast migration along cellular substrates in the developing porcine brain. Stem Cell Rep. *17*, 2097–2110.

Sanai, N., Nguyen, T., Ihrie, R.A., Mirzadeh, Z., Tsai, H.H., Wong, M., Gupta, N., Berger, M.S., Huang, E., García-Verdugo, J.M., et al. (2011). Corridors of migrating neurons in the human brain and their decline during infancy. Nature *478*, 382–386.

Sanai, N., Tramontin, A.D., Quiñones-Hinojosa, A., Barbaro, N.M., Gupta, N., Kunwar, S., Lawton, M.T., McDermott, M.W., Parsa, A.T., Manuel-García Verdugo, J., et al. (2004). Unique astrocyte ribbon in adult human brain contains neural stem cells but lacks chain migration. Nature *427*, 740–744.

Sawamoto, K., Hirota, Y., Alfaro-Cervello, C., Soriano-Navarro, M., He, X., Hayakawa-Yano, Y., Yamada, M., Hikishima, K., Tabata, H., Iwanami, A., et al. (2011). Cellular composition and organization of the subventricular zone and rostral migratory stream in the adult and neonatal common marmoset brain. J. Comp. Neurol. *519*, 690–713.

Shinmyo, Y., Saito, K., Hamabe-Horiike, T., Kameya, N., Ando, A., Kawasaki, K., Duong, T.A.D., Sakashita, M., Roboon, J., Hattori, T., et al. (2022). Localized astrogenesis regulates gyrification of the cerebral cortex. Sci. Adv. *8*, eabi5209.

Sugiyama, A., Nakamura, Y., Akie, Y., Saito, H., Izumi, Y., Yamazaki, H., Kaneko, N., and Itoh, K. (2011). Microminipig, a Non-rodent Experimental Animal Optimized for Life Science Research: In Vivo Proarrhythmia Models of Drug-Induced Long QT Syndrome: Development of Chronic Atrioventricular Block Model of Microminipig. J. Pharmacol. Sci. *115*, 122–126.

Takasu, M., Tsuji, E., Imaeda, N., Matsubara, T., Maeda, M., Ito, Y., Shibata, S., Ando, A., Nishii, N., Yamazoe, K., and Kitagawa, H. (2015). Body and major organ sizes of young mature microminipigs determined by computed tomography. Lab. Anim. *49*, 65–70.

Takeishi, K., Kawaguchi, H., Akioka, K., Noguchi, M., Arimura, E., Abe, M., Ushikai, M., Okita, S., Tanimoto, A., and Horiuchi, M. (2018). Effects of Dietary and Lighting Conditions on Diurnal

Locomotor Activity and Body Temperature in Microminipigs. In Vivo (Athens, Greece) *32*, 55–62.

Torrijos-Saiz, L.I., Freixes, J., Desfilis, E., Medina, L., Sawamoto, K., García-Verdugo, J.M., and Herranz-Pérez, V. (2025). Cellular Organization and Migration Pathways of the Ventricular–Subventricular Zone in the Juvenile Swine Brain (*Sus scrofa domesticus*). J. Comp. Neurol. *533*, e70070.

Wang, W., Yin, C., Wen, S., Liu, Z., Wang, B., Zeng, B., Sun, L., Zhou, X., Zhong, S., Zhang, J., et al. (2024). DCX knockout ferret reveals a neurogenic mechanism in cortical development. Cell Rep. *43*, 114508.

Yun, S.P., Kim, D.H., Ryu, J.M., Park, J.H., Park, S.S., Jeon, J.H., Seo, B.N., Kim, H.-J., Park, J.-G., Cho, K.-O., and Han, H.J. (2011). Magnetic resonance imaging evaluation of Yukatan minipig brains for neurotherapy applications. Lab. Anim. Res. *27*, 309–316.

Bressan, C., Pecora, A., Gagnon, D., Snapyan, M., Labrecque, S., De Koninck, P., Parent, M., and Saghatelyan, A. (2020). The dynamic interplay between ATP/ADP levels and autophagy sustain neuronal migration in vivo. Elife *9*, e56006.

Stem Cell Reports, Volume *21*

Supplemental Information

Cytoarchitecture of neurogenic niche and neuroblast clusters in the postnatal microminipig brain

Daijiro Kojima, Masato Sawada, Taisei Ishimaru, Nodoka Ito, Shinichiro Tateyama, Kazuhide Adachi, Hiroaki Kawaguchi, Noriaki Satake, Vicente Herranz-Pérez, José Manuel García-Verdugo, Yuichi Hirose, Nobuhiko Ohno, Naoko Kaneko, and Kazunobu Sawamoto

Supplementary Information

Cytoarchitecture of neurogenic niche and neuroblast clusters in the postnatal microminipig brain

Daijiro Kojima, Masato Sawada, Taisei Ishimaru, Nodoka Ito, Shinichiro Tateyama, Kazuhide Adachi, Hiroaki Kawaguchi, Noriaki Satake, Vicente Herranz-Pérez, José Manuel García-Verdugo, Yuichi Hirose, Nobuhiko Ohno, Naoko Kaneko, Kazunobu Sawamoto

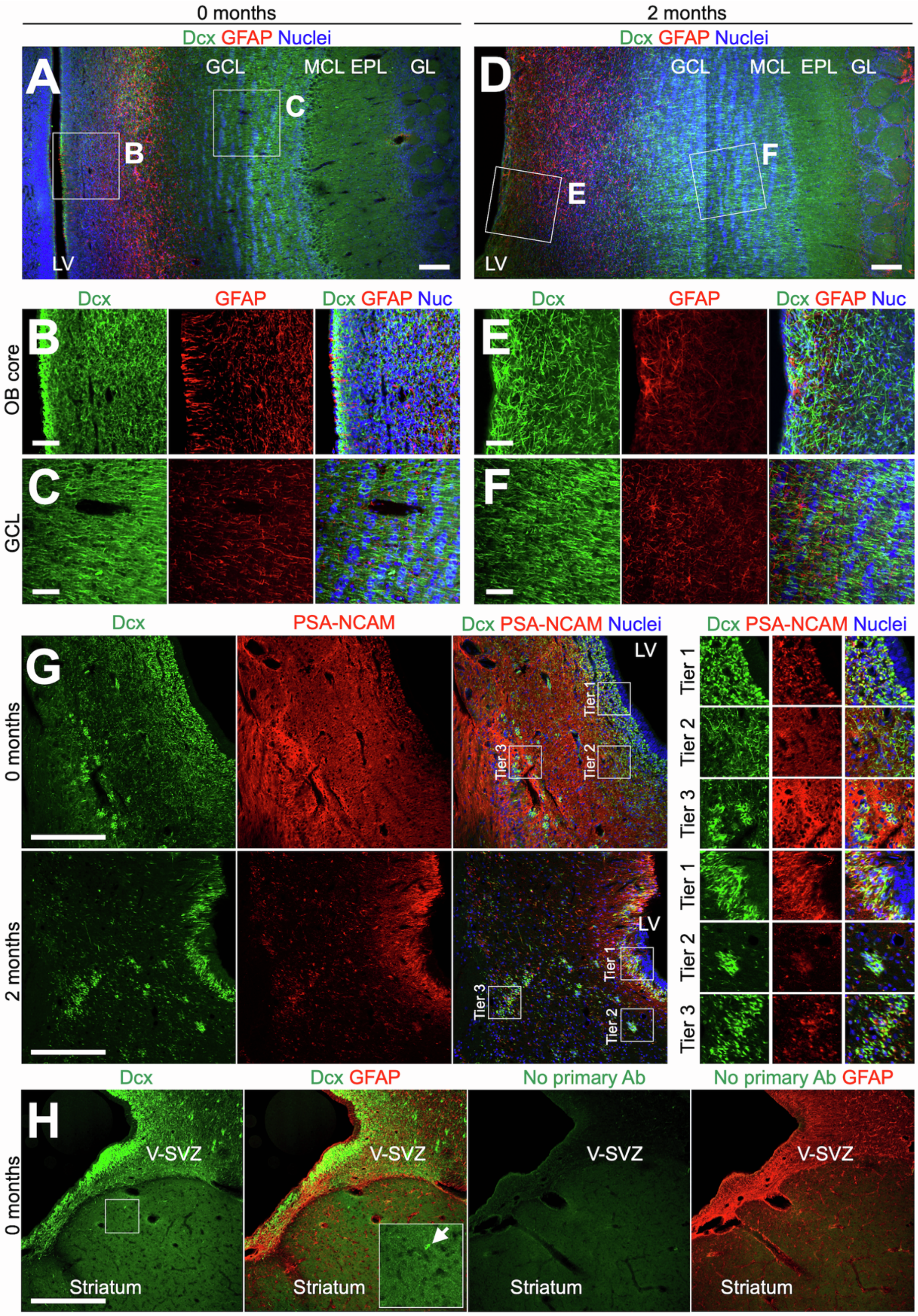

Figure S1. Cytoarchitecture of the OB in postnatal microminipigs, related to Figure 1.

A-F: Representative images of the coronal OB sections in 0-month-old (A-C) and 2-month-old (D-F) microminipigs stained for Dcx (green) and GFAP (red). Nuclei were stained with Hoechst 33342 (blue).

G: Representative images of the coronal V-SVZ sections from 0-month-old (upper panels)

and 2-month-old (lower panels) microminipigs stained for Dcx (green) and PSA-NCAM (red). Nuclei were stained with Hoechst 33342 (blue). Boxed areas are shown at higher magnification in the right panels.

H: Representative coronal sections of the V-SVZ from 0-month-old microminipigs stained for Dcx (green) and GFAP (red) (left two panels), or processed without primary antibodies (green) (right two panels). The boxed area is magnified in the second panel. The arrow indicates a Dcx+ cell in the striatum.

LV, lateral ventricle; GCL, granule cell layer; MCL, mitral cell layer; EPL, external plexiform layer; GL, glomerular layer; V-SVZ, ventricular-subventricular zone.

Scale bars: A, D, 1 cm; B, C, E, F, G, H, 200 μm.

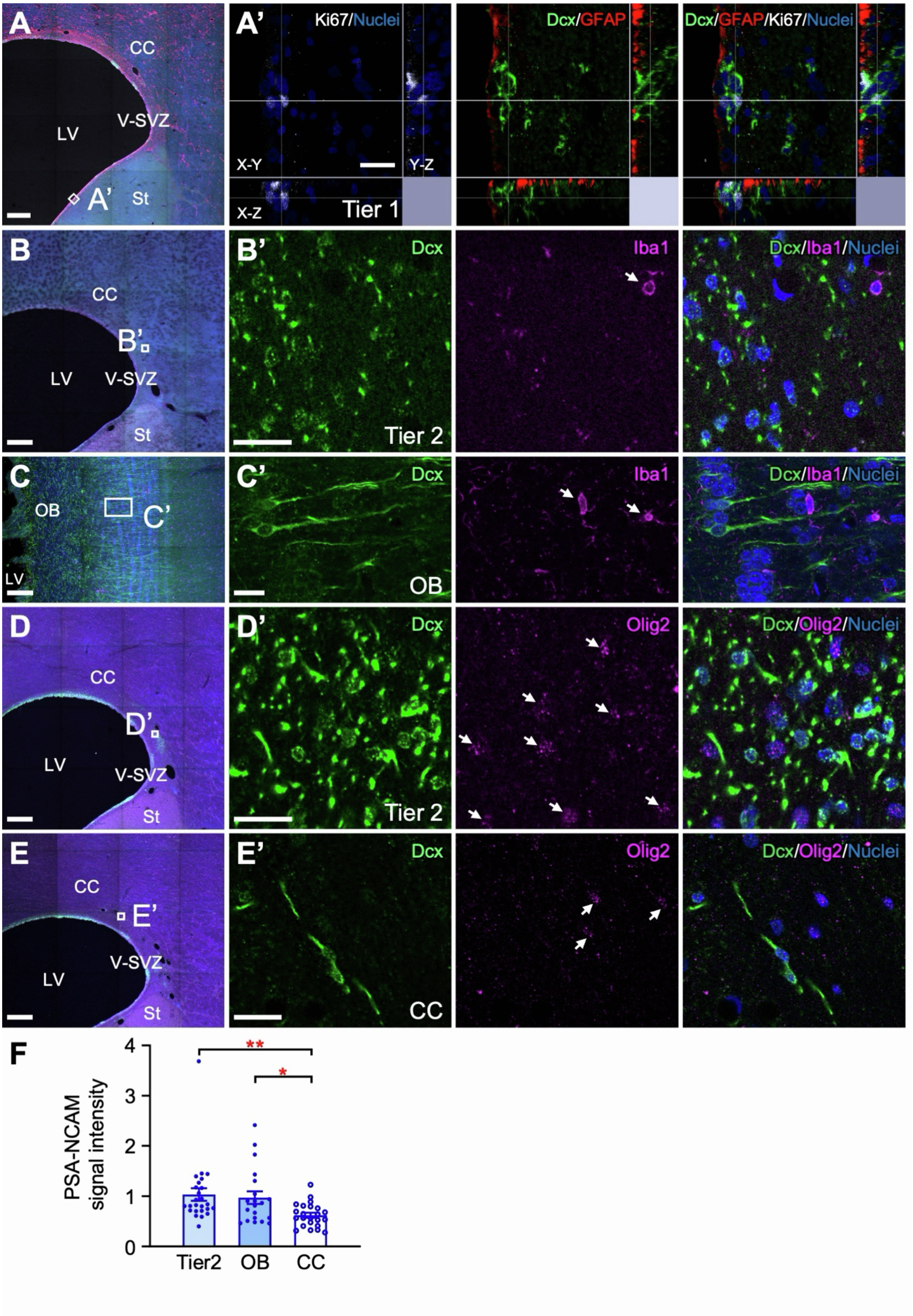

A
CC
LV
V-SVZ
A'
St
A'
Ki67/Nuclei
X-Y
Y-Z
X-Z
Tier 1
Dcx/GFAP
Dcx/GFAP/Ki67/Nuclei
B
CC
B'
LV
V-SVZ
St
B'
Dcx
Tier 2
Iba1
Dcx/Iba1/Nuclei
C
OB
C'
LV
C'
Dcx
OB
Iba1
Dcx/Iba1/Nuclei
D
CC
D'
LV
V-SVZ
St
D'
Dcx
Tier 2
Olig2
Dcx/Olig2/Nuclei
E
CC
E'
V-SVZ
LV
St
E'
Dcx
CC
Olig2
Dcx/Olig2/Nuclei
F
PSA-NCAM signal intensity
0
1
2
3
4
**
*
Tier2
OB
CC

Figure S2. Characterization of Dcx+ cells, related to Figure 2.

A: Confocal images of coronal brain sections from an adult microminipig immunostained for Dcx (green), GFAP (red), and the proliferation marker Ki67 (white). Nuclei were stained with Hoechst 33342 (blue). The boxed area is shown at higher magnification in A', with orthogonal views displayed as x-y (bottom) and x-z (right) panels.

B-C: Confocal images of the adult microminipig coronal brain sections immunostained for neuroblast marker Dcx (green) and microglial marker Iba1 (magenta). Nuclei were stained with Hoechst 33342 (blue). B shows a higher magnification image of the boxed area in A. Iba1+ cells (arrows) did not co-express Dcx in either tier 2 (A-B) or olfactory bulb (C).

D-E: Confocal images of the adult microminipig coronal brain sections immunostained for Dcx (green) and oligodendrocyte lineage marker Olig2 (magenta). Nuclei were stained with Hoechst 33342 (blue). E shows a higher magnification image of the boxed area in D. Olig2+ cells (arrows) did not co-express Dcx in either tier 2 (D-E) or corpus callosum (F). OB, olfactory bulb; CC, corpus callosum.

F: Quantification of PSA-NCAM signal intensity in Dcx+ cells located in tier 2, the corpus callosum (CC), and the olfactory bulb (OB). To normalize for background staining, the mean cytoplasmic PSA-NCAM (magenta) signal intensity of each cell, measured using Fiji, was divided by the corresponding nuclear signal intensity. PSA-NCAM signal intensity was significantly lower in Dcx+ cells in the CC compared with those in tier 2 and the OB (Kruskal-Wallis test). *$P<0.05$, **$P<0.01$.

Scale bars, 500 μm: A, B, D, E; 200 μm: C; 20 μm: A', B', C', D', E'.

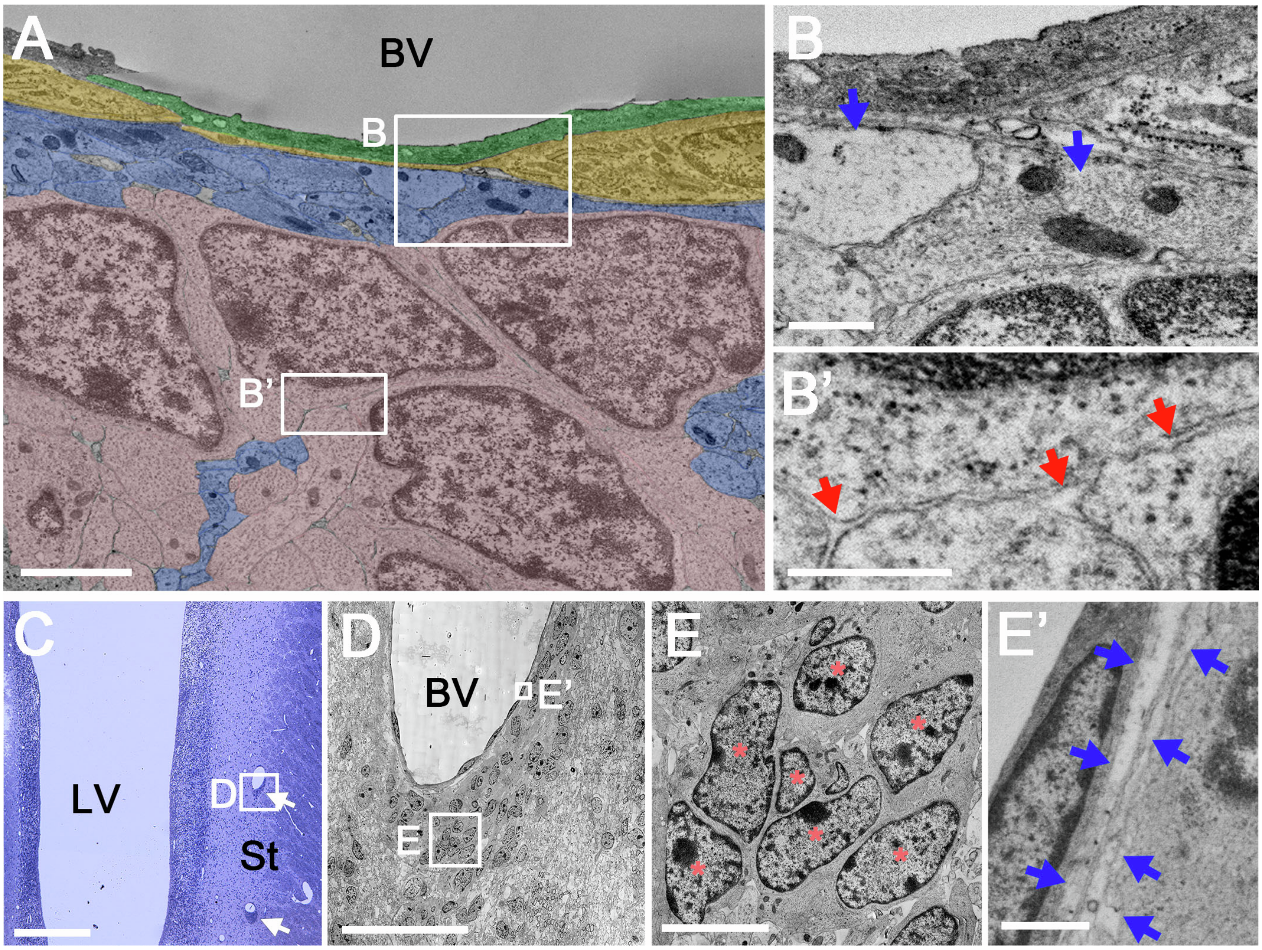

Figure S3. Ultrastructural analysis of neuroblast-blood vessel and neuroblast-neuroblast interaction, related to Figure 3.

A-B': A large neuroblast cluster located in tier 3 of the dorsolateral V-SVZ. The cluster is closely associated with vasculature (A, pink: neuroblasts, blue: astrocytes, green: endothelium, orange: pericytes). Higher magnification views of the boxed areas in A are shown in B and B'. Astrocytic endfeet (blue arrows) are interposed between the blood vessel and the adjacent neuroblasts (B). Neuroblasts are densely packed, forming narrow intercellular spaces (B', red arrows).

C-E': Neuroblast clusters in the striatum. A semi-thin toluidine blue-stained section (C) shows large neuroblast clusters (white arrows) surrounding blood vessels in the striatal parenchyma. A low magnification TEM image of the boxed area in C is shown in (D). Higher magnification views of the boxed areas in D are shown in E and E'. Neuroblasts are marked with pink asterisks (E). Thin astrocytic endfeet between the blood vessel and neuroblasts are indicated with blue arrows (E').

Abbreviations: LV, lateral ventricle; St, striatum; BV, blood vessel. Scale bars, 500 μm: C; 50 μm: D; 5 μm: E; 2 μm: A; 1 μm: E'; 0.5 μm: B, B'.

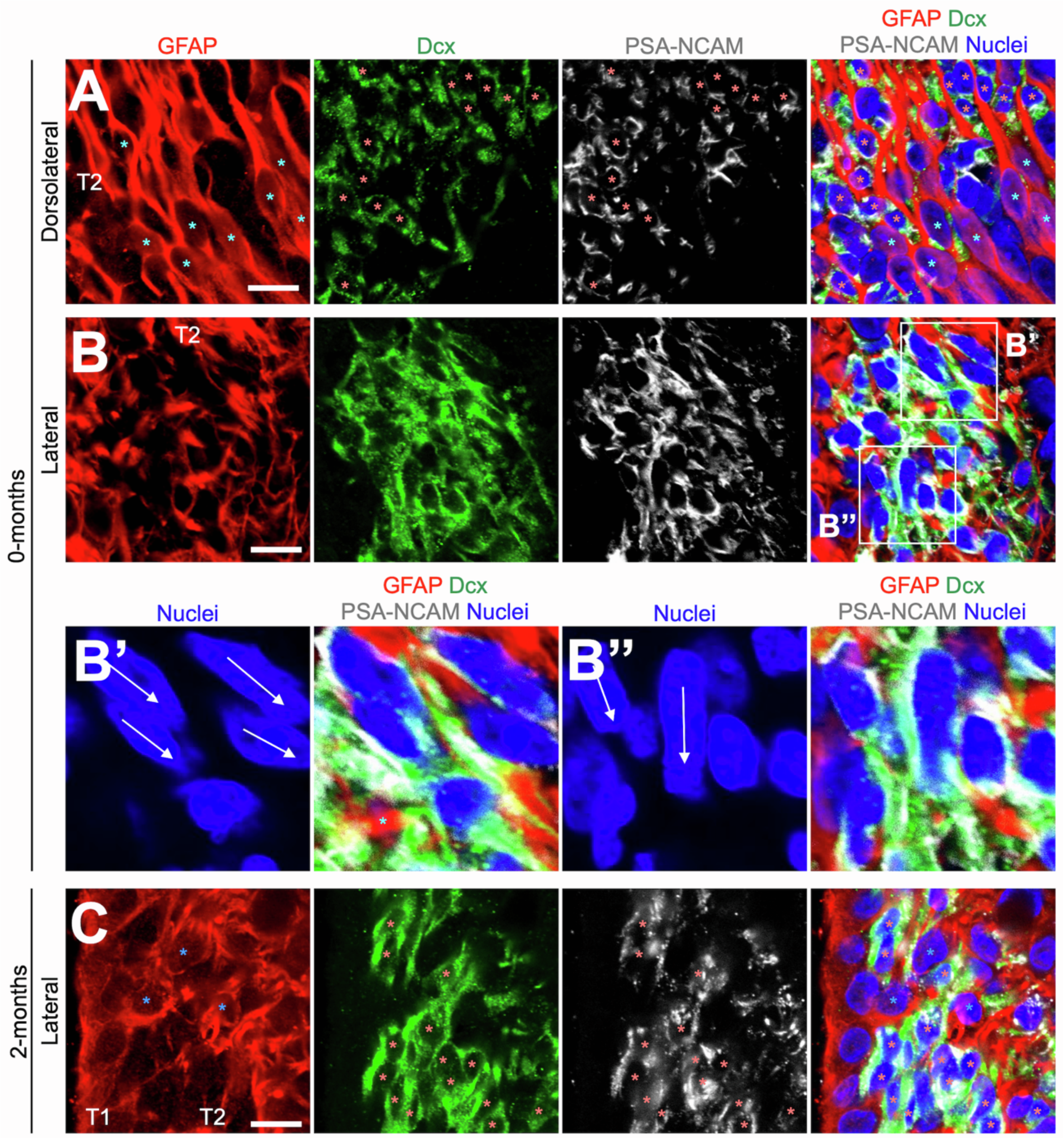

Figure S4. Identification of cell types in tier 2 of 0-month-old and 2-month-old microminipigs, related to Figures 3 and 4.

A-C: Representative images of the coronal V-SVZ sections from 0-month-old (A-B'') and 2-month-old (C) microminipigs stained for Dcx (green), GFAP (red), and PSA-NCAM (white). Nuclei were stained with Hoechst 33342 (blue). Light blue (A), pink (A, C), and dark blue (C) asterisks indicate GFAP+ radial glia, Dcx+PSA-NCAM+ neuroblasts, and GFAP+ astrocytes, respectively. Arrows (B' and B'') indicate cellular orientation. Boxed areas in (B) are shown at higher magnification in (B') and (B'').

T1, tier 1; T2, tier 2. Scale bars, 10 μm.

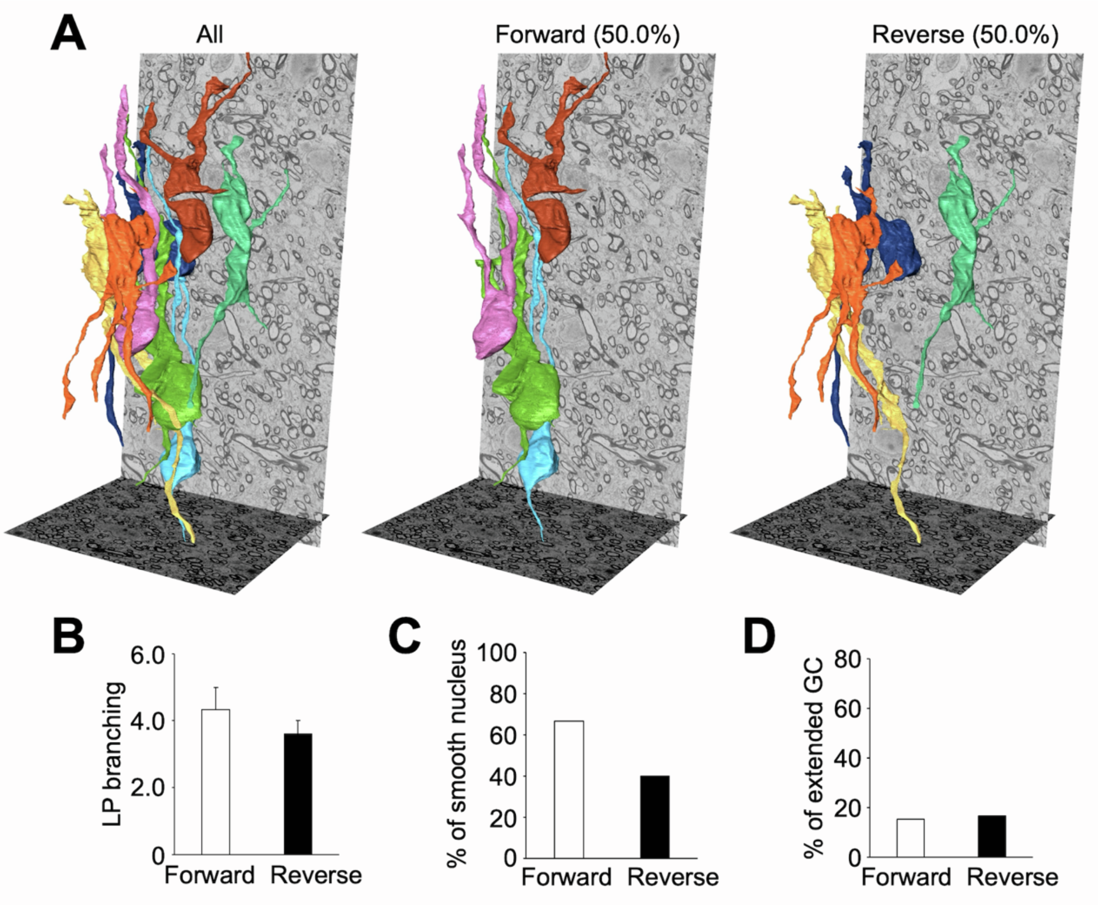

Figure S5. Three-dimensional morphology of neuroblast clusters located farther from blood vessels in tier 3

A: Representative SBF-SEM images of cluster of migratory neuroblasts located farther from blood vessels in tier 3 of the V-SVZ in 2-month-old microminipigs. The directions of neuroblasts are defined based on the directionality of blood vessel-associated neuroblasts shown in Figure 7A.

B: Number of leading process branches in forward- and reverse-migrating neuroblasts.

C: Proportion of cells with a smooth nucleus in forward- and reverse-migrating neuroblasts.

D: Proportion of cells with an extended growth cone in forward- and reverse-migrating neuroblasts.

Table S1. Ultrastructural characteristics of cell types in the postnatal V-SVZ of microminipigs.

	Neuroblasts	Radial glial cells	Astrocytes	Ependymal cells
**Location**	Tiers 1-3	0 months: tier 1/tier 2 (inner)<br>2 months: (-)	0 months: tiers 1/2 (few)<br>tier 3 (many)<br>2 months: tiers 1-3	Ventricular surface
**Contour**	Smooth	Elongated, Smooth	Irregular	Frequent adherens junctions<br>Interdigitated
**Cytoplasm**	Scant<br>Free ribosomes<br>Microtubules	Intermediate filaments<br>9+0 cilium	Light<br>Intermediate filaments<br>9+0 cilium	Basal bodies<br>Multiple 9+2 cilia<br>Apical mitochondria
**Nucleus**	Elongated, Small	Irregular<br>Occasionally invaginated	Irregular, Electron lucent<br>Occasionally invaginated	Spherical, Large

Supplementary Methods

Immunohistochemistry

Immunohistochemistry was performed as described previously with slight modification (Akter et al., 2020). Briefly, the microminipigs were deeply anesthetized by intramuscular injection of a mixture of 0.04 mg/kg medetomidine, 5 mg/kg ketamine hydrochloride, and 0.2 mg/kg midazolam or by inhalation of isoflurane, and then fixed by transcardiac perfusion with 4% paraformaldehyde (PFA) in 0.1 M phosphate buffer (PB) (pH 7.4). The brains were postfixed in the same fixative at 4 ºC. 70-μm-thick coronal sections were cut using a vibratome (VT-1200S, Leica). Coronal sections (70 μm-thick) from the olfactory bulb (OB) to anterior V-SVZ regions were incubated for 40 min at RT in blocking solution (10% normal donkey serum and 0.4% TritonX-100 in PBS), overnight at 4 ºC with the primary antibodies, and 3 h at RT with AlexaFluor-conjugated secondary antibodies (1:1,000, Invitrogen) in the blocking solution. The following primary antibodies were used: goat anti-doublecortin (Dcx) (1:500, Santa Cruz Biotechnology); mouse anti-glial fibrillary acidic protein (GFAP) monoclonal (1:500, Sigma). Nuclei were stained with Hoechst 33342 (1:5,000, Sigma).

Transmission Electron Microscopy

Neonatal and 2-month-old microminipig brains were fixed by transcardiac perfusion with 2.5% glutaraldehyde (GA) and 2% PFA in 0.1 M PB (pH 7.4) and postfixed in the same fixative at 4 ºC. Three hundred-μm-thick coronal sections from OB to anterior V-SVZ regions were cut using a vibratome (VT-1200S, Leica), followed by treatment with 2% OsO_4 in the same buffer for 2 h at 4°C and treatment with 1% uranyl acetate/50% ethanol for 1 h at 4°C. The sections were then dehydrated in a graded ethanol series, placed in propylene oxide, and embedded in Durcupan resin. Semi-thin sections (2.0 μm-thick) were sequentially cut using an ultramicrotome (UC6, Leica Microsystems) with a diamond knife (histo, DiATOME). The sections were stained with 1% toluidine blue solution, and those of interest were chosen under a light microscope CX23 (Olympus). Serial ultra-thin sections (60-70 nm-thick) were cut from the embedded semi-thin sections using an ultramicrotome (UC6, Leica Microsystems) with a diamond knife (SYM2045, SYNTEK), and then stained with 2% uranyl acetate in distilled water for 15 min and with modified Sato's lead solution for 5 min. Sections were examined using a transmission electron microscope (JEM-1400Plus; JEOL, Tokyo, Japan). Images at 8,000× magnification were stitched using Shot Meister software (TEMography.com) (Figure 3B-D, F, G, J-L, N-Q, W-Y', Figure 4B, D-G', J-L', Q).

For quantification of neuroblast contact with surrounding cells (Figures 3, 4), whole-cell images were acquired at a magnification of 8,000 × . Only membrane segments

in direct contact with other cells or structures were analyzed. The segments were classified according to the contacting element—radial glia, neuroblasts, astrocytes, blood vessels, other cells, or no contact—and their length was measured using Fiji software (National Institutes of Health, USA). The percentage of membrane length for each category relative to the total measured membrane was then calculated.

Serial Block-Face Scanning Electron Microscopy

Two-month-old microminipig brains were fixed by transcardiac perfusion with 2.5% GA and 2% PFA in 0.1 M PB (pH 7.4) and postfixed in the same fixative at 4 ºC. The V-SVZ tissue blocks dissected from the 300-μm-thick coronal sections were treated with 2% OsO_4 and 1.5% potassium ferricyanide in PBS for 1 h at 4°C, 1% thiocarbohydrazine for 20 min at RT, 2% aqueous OsO_4 for 30 min at RT, and lead aspartate solution for 30 min at 65°C. The samples were then dehydrated in a graded ethanol series, treated with dehydrated acetone, and embedded in Durcupan resin containing Ketjenblack powder.

Serial image sequences were acquired at a resolution of 6 nm/pixel and at 80 nm steps. Sequential images were processed using FIJI. Orientation of each neuroblast was determined by the direction of the leading process and the position of the centrosome. Growth cone extension and collapse are defined as a distal membrane expansion that is more than three times larger or smaller, respectively, than the diameter of the leading process.

Statistics

The Shapiro-Wilk test was used to assess normality and equal variances between groups. When data did not meet the assumptions of normality or equal variance, the Steel-Dwass test or Kruskal-Wallis test was applied. For comparisons between two independent groups in the SBF-SEM analyses (Figure 7), Fisher’s exact test or an unpaired *t*-test was used.
